# Analysis of the *P1* promoter in response to UV-B radiation in allelic variants of high-altitude maize

**DOI:** 10.1186/1471-2229-12-92

**Published:** 2012-06-15

**Authors:** Sebastián Pablo Rius, Erich Grotewold, Paula Casati

**Affiliations:** 1 Centro de Estudios Fotosintéticos y Bioquímicos (CEFOBI), Universidad Nacional de Rosario, Suipacha 531, Rosario, Argentina; 2 Plant Biotechnology Center, The Ohio State University, Columbus, OH, 43210, USA; 3 Department of Plant Cellular and Molecular Biology, The Ohio State University, Columbus, OH, 43210, USA

**Keywords:** UV-B radiation, Maize landraces, Promoters, P1 transcription factor

## Abstract

**Background:**

Plants living at high altitudes are typically exposed to elevated UV-B radiation, and harbor mechanisms to prevent the induced damage, such as the accumulation of UV-absorbing compounds. The maize R2R3-MYB transcription factor P1 controls the accumulation of several UV-B absorbing phenolics by activating a subset of flavonoid biosynthetic genes in leaves of maize landraces adapted to high altitudes.

**Results:**

Here, we studied the UV-B regulation of *P1* in maize leaves of high altitude landraces, and we investigated how UV-B regulates P1binding to the *CHS* promoter in both low and high altitude lines. In addition, we analyzed whether the expansion in the *P1* expression domain between these maize landraces and inbred lines is associated to changes in the molecular structure of the proximal promoter, distal enhancer and first intron of *P1*. Finally, using transient expression experiments in protoplasts from various maize genotypes, we investigated whether the different expression patterns of *P1* in the high altitude landraces could be attributed to *trans*- or *cis*-acting elements.

**Conclusions:**

Together, our results demonstrate that, although differences in *cis*-acting elements exist between the different lines under study, the different patterns of P1 expression are largely a consequence of effects in *trans.*

## Background

Plants, because of their sessile lifestyle, have evolved adaptations to live under diverse environmental variables, including solar radiation. Sunlight contains qualities of light that are essential for photosynthesis, and also provides informational signals that control plant growth and development. At least three different types of photoreceptor systems exist in plant cells, perceiving red/far-red (phytochromes), blue/UV-A (cryptochromes and phototropins), and there is at least one putative UV-B receptor (UVR8), which was recently identified [1].

UV-B (280 to 315 nm) is part of the solar radiation, and therefore plants are inevitably exposed to UV-B. UV photons cause cellular damage by generating DNA photoproducts and through direct damage to proteins, lipids, and RNA [2-4]. Plant responses to UV-B damage include repair [5,6] and avoidance [7,8]. Solar UV-B radiation flux varies over time and location on earth. It is higher at increased elevation, because there is less air mass and greater atmospheric transparency to shorter wavelength radiation, although local climatic conditions, such as cloud cover, can modulate the dosage [9]. UV-B increases are most critical in specific ecosystems, for example, ozone depletion was extensive at a 3420-m site in the Andes Mountains compared with values from equal latitude to the west and east [10]. Consequently, determining the molecular bases for acclimation to higher UV-B fluence is an important factor in sustaining crop yield as the world’s population continues to increase.

Because plants living at high altitudes are typically exposed to elevated UV-B, they are predicted to have mechanisms to prevent damage from this radiation, such as the accumulation of UV-absorbing compounds [7,11] and the use of UV-A photons by photolyase enzymes to repair most UV-B induced DNA damage [2]. The plant epidermis, by virtue of the accumulation of numerous phenolic compounds and cuticular waxes, absorbs 90–99% of solar UV-B radiation. In many flowering plants, flavonoids accumulate in the vacuoles of epidermal cells where they attenuate the UV component of sunlight with minimal absorption of photosynthetically active radiation [11,12].

The *P1* gene encodes an R2R3-MYB transcription factor that regulates the accumulation of a specific group of flavonoids in maize floral tissues, the flavones and the phlobaphenes [13]. P1 controls the accumulation of these pigments by activating a subset of maize flavonoid biosynthetic genes [13-15] and is primarily expressed in floral tissues, including the pericarp, cob glumes, silks and husk tissues. The phlobaphene pigments have been historically used as markers to uncover some of the fundaments of modern genetics. Among the compounds controlled by *P1* are the flavones, important phytochemicals that provide protection against a number of maize pathogens, furnish a powerful UV shield and are significant nutraceutical components of the human diet [16]. Previously, we established that some maize landraces, specifically those adapted to high altitudes, accumulate flavones and express *P1* in leaves and other green tissues in the presence of UV-B, in sharp departure to the floral-organ specific expression domain of *P1* found in most other maize inbred lines [17]. These results suggest the potential for a large P1 allelic diversity as a consequence of growing in diverse environments.

An important challenge is to define the molecular bases that permit acclimation responses of maize to UV-B. Maize races have a complex history, having been derived from multiple open pollinated varieties and transported by people to diverse locations [18]. Maize genotypes exhibit immense allelic diversity, and this represents a fundamental resource for both genetics and breeding. This crop requires a high light environment for good yield [19], and it is safe therefore to assume that indigenous farmers would grow high-altitude maize in sunny locations. Therefore, these indigenous landraces are predicted to have improved UV-B tolerance, reflecting recurrent selection against visible symptoms of UV-B stress.

In this study, we analyzed whether the expansion in the expression domain of *P1* in specific maize landraces that have adapted to high altitude (and hence to higher UV-B levels) can be associated to changes in the molecular structure of the corresponding *P1* alleles, in particular, changes in *cis*-regulatory elements sequences in promoter regions that allow as-yet to be identified regulators to activate *P1* expression in vegetative tissues. To gain insight of the participation of this transcription factor in UV-B regulated synthesis of flavones in maize leaves, we first studied the regulation of *P1* by UV-B in maize leaves of five maize landraces from high altitudes: three from Mexico (Cacahuacintle, Conico Norteño and Arrocillo Amarillo, collected from altitudes between 2200- and 2800-m) and two from the Andes mountains (Mishca from altitudes between 2200-and 2800-m and Confite Puneño from altitudes between 3600- and 3900 m). We then analyzed the molecular structure of the *P1 *promoter alleles in these landraces, and investigated which regulatory motifs may be responsible of the regulation of *P1* by UV-B. Finally, to study if the different expression patterns of *P1* in the high altitude landraces can be attributed to the presence of *trans*- or *cis*-acting elements in the landraces, transient expression analysis using leaf protoplasts from W23 and Mishca, and proximal promoter constructs fused to the luciferase gene reporter were analyzed.

## Results

### *P1* is expressed in maize leaves and is induced by UV-B in maize high altitude landraces

**Figure 1:**
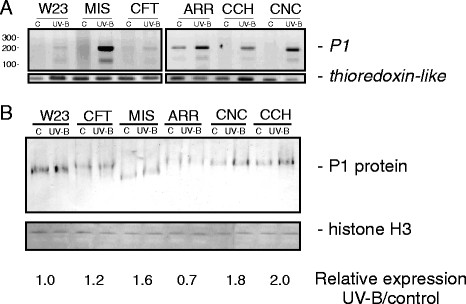
**Regulation of P1 expression by UV-B.** (**A**) *P1* transcript analysis was performed by nested RT-PCR in leaves of W23 and maize landraces after an 8 h-UV-B-treatment or under control conditions in the absence of UV-B. The non-UV-B regulated transcript *thioredoxin-like* transcript was used as a control. (**B**) Western-blot analysis of nuclear leaf protein extracts of W23 and maize landraces after an 8 h-UV-B-treatment or under control conditions in the absence of UV-B using antibodies against P1. 10 μg of nuclear proteins were loaded in each lane. The relative protein levels obtained from the quantification of the western blot bands by densitometry were compared between UV-B and control conditions. MIS: Mishca; CFT: Confite Puneño; ARR: Arrocillo Amarillo; CCH: Cacahuacintle; CNC: Conico Norteño. All experiments were done in triplicate using biological replicates.

Previously, northern blot analysis using a complete *P1* cDNA as a probe identified a transcript induced by UV-B radiation in leaves of high-altitude maize plants, with no signal in mRNAs from W23 leaves [17]. Until then, no *P1* alleles had been reported to be expressed in maize leaves. By the nature of the experiment, however, it is possible that this long probe could have hybridized not only to *P1*, but also to any other transcripts harboring similarity to *P1*. For example, *P1* is a MYB-like transcriptional activator [13]; it is possible that the probe used can recognize transcripts for another MYB regulator. To rule out this possibility, to detect the very low abundance of *P1* transcripts in leaves, and to analyze *P1* regulation by UV-B in the different genetic backgrounds, here we analyzed the expression of *P1* in landraces and inbred lines leaves subjected to UV-B radiation treatments using nested RT-PCR, a significantly more specific and sensitive technique. *P1* expression level was measured in young leaves of five different maize high-altitude landraces and in one low altitude line, W23 (Figure 1A). After 8 hours of UV-B, an important increase in *P1* transcript levels was measured in the five landraces when compared with levels in plants under control conditions in the absence of UV-B, which were almost undetectable under this condition (Figure 1A). In contrast, the W23 inbred line showed very low and similar *P1* transcript levels under both conditions. 

Furthermore, P1 protein levels were analyzed by western blots, using polyclonal antibodies against P1 [20], in nuclear extracts from leaves of a low altitude inbred line (W23) and the five landraces, under control and UV-B conditions. The densitometric quantification of the western blots showed increases of P1 protein levels in the high altitude landraces (with the exception of Arrocillo) after the UV-B treatment (Figure 1B). Consistent with the RT-PCR results (Figure 1A), W23 did not show changes in *P1* levels after irradiation with supplemental UV-B (Figure 1B). Thus, mRNA changes by UV-B parallels the protein changes measured. It is interesting that, despite the low levels of *P1* transcript measured in leaves, in some cases almost undetectable as determined by nested RT-PCR, it was possible to distinguish the presence of the protein in nuclear extracts in all samples at different levels.

### UV-B increases the binding of P1 to the *CHS* promoter in high altitude landraces

An 8 h-long UV-B-treatment can induce the expression of enzymes of the flavonoid pathway in maize, including chalcone synthase (CHS) [21]. Maize has two CHS genes, C2 and WHP [22]. Thus, we analyzed the association of *P1* to the promoter region of *C2* in one high-altitude landrace, Confite Puneño (CFT), and in the W23 low altitude line under control conditions (without UV-B) and after an 8 h UV-B treatment.

**Figure 2:**
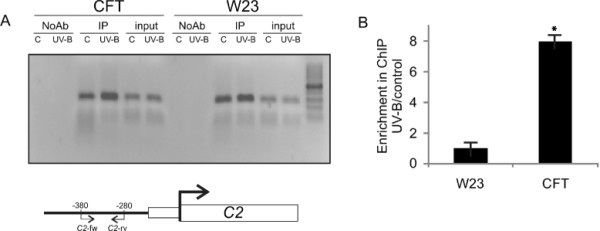
**Chromatin immunoprecipitation (ChIP) assay using antibodies against P1.** ChIP analysis was done by both PCR (**A**, 25 cycles) and qPCR (**B**) using mature leaves from B73 and Confite Puneño (CFT) after an 8 h UV-B treatment or under control conditions in the absence of UV-B. Primers designed to amplify the *CHS* promoter are listed in Table S1. IP, ChIPed sample; NoAb, No-antibody control; input: input DNA. All experiments were done in triplicate using biological replicates. Asterisks denote statistical differences applying Student’s t test (*P* < 0.05).

Chromatin immunoprecipitation (ChIP) analyses were done using the polyclonal antibodies against P1(αP1) [20] using control and UV-B treated samples from Confite and W23 maize leaves, and enrichment after immunoprecipitation was analyzed by PCR using primers specific for the *C2* promoter region (Figure 2A). To evaluate nonspecific binding, the PCR reaction was done with samples incubated without an antibody; all ChIPed samples were also analyzed in parallel with total DNA from sonicated nuclei to evaluate the selective recovery of gene segments. The percentage of DNA recovered relative to the DNA input when experiments were done in the absence of antibodies was always lower than 5% of the DNA recovered when specific antibodies were used (not shown). After the UV-B treatment, *C2* promoter sequences in Confite plants were increased in the fractions immunoprecipitated with αP1; in contrast, similar levels of immunoprecipitate were measured in W23 samples with or without the UV-B treatment (Figure 2B). No enrichment was detected after the UV-B treatment in the control without antibody (Figure 2A) for either Confite or W23 samples. Therefore, there is a considerable increase in P1 binding to the *CHS* promoter region of Confite plants exposed under UV-B light, and this change does not occur in the inbred line W23 (Figure 2B)*.* From these results, we propose that the increased expression of *P1* in CFT and other high-altitude races results in P1 regulating *C2* expression during UV-B exposure in leaves of the maize landrace Confite. A similar mechanism is likely involved in the regulation of the expression of other genes encoding flavonoid biosynthesis enzymes. 

### Variable P1 copy number in maize landraces

**Figure 3:**
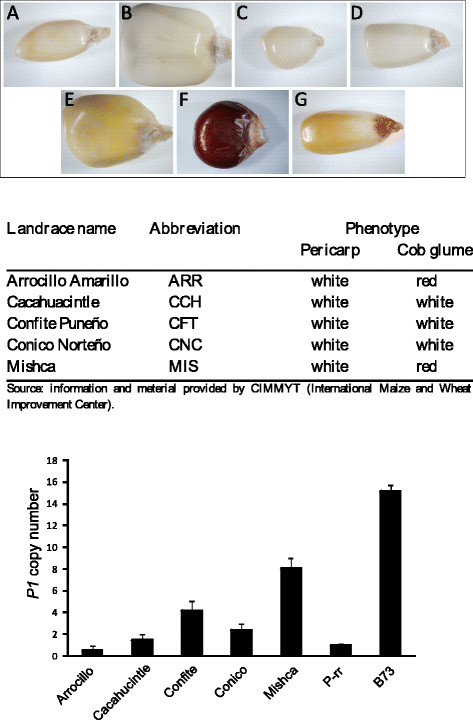
**Kernel images,**_***p1*alleles**_**and estimation of the copy number of*P1*genes in the different high altitude landraces.** Individual kernel images from the different high altitude landraces analyzed, B73 and *P1-rr*; **A**: Arrocillo Amarillo; **B**: Cacahuacintle; **C**: Confite Puneño; **D**: Conico Norteño; **E**: Mishca (white pericarp); **F**: *P1-rr* and **G**: B73. The determination of the *P1* copy number was performed by quantitative PCR analysis. The *P-rr* allele was used as a reference, and the *ACT-1* gene was used as control of DNA template. Genomic DNA was quantified and each reaction was normalized using the *C*_t_ values corresponding to the *Act-1* transcript. Location of primers used in shown in Additional file 1: Figure S1B. All measurements were done in triplicate using biological replicates.

The number of *P1* copies varies depending on the maize genotype. To investigate the nature of the *P1* allele in the high altitude maize landraces, we determined the copy number of the different alleles of the high altitude landraces by quantitative PCR [23]. Genomic DNA from the single copy *P1-rr* allele was used as a reference, and the *P1-wr* cluster from B73 as an example of the multi-copy *P1* allele. The primers used are specific for *P1*, and do not hybridize on the *P2* gene, an ortholog of *P1* that is not involved in phlobaphene pigmentation [24]. Figure 3A shows the different high altitude landraces, inbred line B73 and *P1-rr* kernels at the same magnification, presenting different color or colorless of the pericarp and the stalk corresponding with the color of the cobs from which genomic DNA was extracted to analyze the *P1* copy number. 

Figure 3B shows that Arrocillo has only 1 copy of the *P1* gene, Cacahuacintle has 2 copies, Confite has 4, Conico has 3 and Mishca has 8 copies of the *P1* gene. On the other hand, our assays show that B73 has 15 *P1* copies (Figure 3), which is close to the eleven copies of *P1* reported by sequencing different BAC clones from this inbred line by Goettel and Messing [25]. Our results show that the landraces under study have a variable number of *P1* copies, suggesting that the UV-B regulation of this gene in the landraces in vegetative tissues is unlikely to be linked to the structure of the *P1* cluster.

### Analysis of *P1* promoters from maize lines and landraces

**Figure 4:**
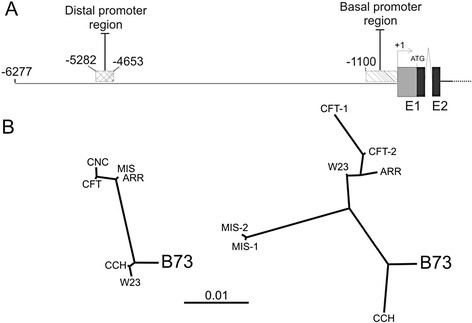
**Schematic representation of*P1*promoter regions and radial trees of non- coding*P1*sequences.** (**A**) Schematic representation of distal (−5282 to −4653) and basal (−1100 to +1) promoter regions (dashed boxes), within the *P1* gene. Distances are represented at scale. (**B**) Phylograms (radial tree) of both promoter regions of the *P1* gene in different landraces, B73 and W23 inbred lines. ARR: Arrocillo Amarillo, CCH, Cacahuacintle; CNC: Conico Norteño; CFT: Confite Puneño; MIS: Mishca.

Because we found that high altitude landraces show *P1* induction by UV-B, and higher expression in leaves compared to the low altitude lines B73 and W23, we then cloned and compared *P1* promoter sequences from the different landraces to analyze the presence of putative regulatory sequences that may be responsible for the differential patterns of expression. These sequences include a distal enhancer and the proximal promoter located between −5282 to −4653 and −1100 to +1, respectively; and the 1^st^ intron region. The DNA sequence of the *P1* promoter (Figure 4A) from different alleles*,* including *P1-wr* and *P1-rr*, was previously reported.

We first cloned and analyzed promoter regions which cover ~1.1kbp upstream the *P1* transcription start site*,* and which include the basal promoter and part of the proximal enhancer described [26] from four of the high altitude landraces (Arrocillo, Confite, Cacahuacintle and Mishca). For comparison, we included in our analysis promoter regions from B73 and W23 lines. We obtained two clones with distinct promoter sequences from Confite and Mishca, and one from Arrocillo and Cacahuacintle, a good correlation, but not the complete set, of the copy number present in the genome (Figure 3). It is however possible that the different copies might be identical in the regions analyzed, or that the primers used to amplify the promoter regions may not hybridize with all the different copies in each landrace.

When the proximal promoter sequences of all lines was compared, a total of 19 indels were identified in this ~1.1kbp region, with single-nucleotide indels accounting for more than half (59.1%), followed by two nucleotide indels (9.1%, Additional file 1: Figure S1A). Thus, more than 68% of all the indels identified were less than 2 bps in length. Moreover, in the middle region of 783 bps sequence analyzed (from −1020 to −237, Figure 4A); there are no indels longer than 2 bps.

It is interesting to note that there is also a relatively high frequency of indels ≥ 15 bases, that include four indels of 18, 19, 24 and 36 bps in length located in the region extending from −253 to +1 (referred to the B73 promoter); and a 15 bps indel is located at −1084. With exception of the 19 bps indel, these long indels are multiples of 3 bps. The 36 bps insertion is exclusively present in the B73 line; the 24 bps insertion is absent in Mishca; the 19 bps insertion is absent in B73 and Cacahuacintle, and the 18 bps insertion is absent only in Cacahuacintle. The 15 bps indel, positioned at −1084 bps (referred to B73), is absent in W23, Arrocillo, and Confite. The sequences included in these long indels do not have any characterized box linked to UV-B or light response elements described in other promoters [27,28]. However, the absence or presence of these indels could affect the relative distance of *cis*-regulatory element to others boxes, which may be involved not only in response to light but also in the modulation of *P1* expression levels.

The high indel frequency observed in maize, and specifically in the cloned promoter regions of the landraces analyzed, may lead to PCR failure when primers are designed from a sequence derived from a genotype different to B73 from the one used in the DNA amplification experiment [29]. This could at least partially explain why we were not able to amplify by PCR the proximal promoter region from Conico.

We also analyzed the sequences described as a distal *P1* enhancer that covers ~630 bp, and it is located 4.6kbp upstream the transcription start site (region from −5282 to −4653, Figure 4A). This sequence includes a sub-fragment of 458 bp of the P1.2 enhancer described by Sidorenko and Peterson [26]. The primers used to clone these sequences are listed in Additional file 2: Table S1. We were able to get only one sequence from this region for each of the five landraces, and of the inbred lines B73 and W23. In this region, we identify three elements that have been described to correspond to binding sites for transcription factors in response to UV-B [28]: a MRE^*CHS*^ core (ACCTA sequence), which is present only in the landraces with the exception of Cacahuacintle (CACGT sequence), and two UVBox^*ANAC13*^boxes (CCAAG sequence) and one core corresponding to this box (CAAG) (Additional file 1: Figure S1B). Both 5 bps long UVBox^*ANAC13*^ boxes are present in all analyzed promoter distal sequences, and are separated by only 5 bps with the sequence AG^C^/_A_CC. The UVBox core sequence, present in all the sequences analyzed, is located nine nucleotides upstream an UVBox^*ANAC13*^ box (Additional file 1: Figure S1B). These UVBoxes^*ANAC13*^ were found significantly enriched in late induced UV-B genes [28].

We also cloned and analyzed the first *P1* intron from B73, W23 and 4 landraces. This intron is usually very small (less than 200 bps) in MYB transcription factors [30]. Within the 120 bps of this intron, we only identified five and one nucleotide changes with respect to B73 and W23 in Confite and Mishca, respectively (Additional file 1: Figure S1C). The remaining sequence resulted identical between them and respect to B73 and W23.

The six 1^st^ intron sequences harbor an ACE^CHS^ core, located 33 bps from the start of the intron (CACGT sequence). In the case of Mishca, two of the three changes are located at each side of the ACE^*CHS*^ core (Additional file 1: Figure S1C).

Recently, sequencing data has demonstrated that the maize genome exhibits variable levels of genetic diversity depending on the lines under comparison. On average, the frequency of single nucleotide polymorphisms between two maize inbred lines is approximately 1 substitution per 100 bases [31]. In our case, the comparison of both the proximal and the distal promoter regions, and the 1^st^ intron of *P1* between inbred lines and high altitude landraces showed some minor, but possibly still important, sequence variation that may be responsible for the differences observed in *P1* expression patterns.

Together, our analysis of the *P1*promoter and 1^st^ intron nucleotide sequences in the high altitude landraces and inbred lines suggests that there may be a selective pressure over some regulatory regions that may explain the different UV-B regulation of *P1*.

### Nucleotide diversity in *P1* non-coding regions

Finally, nucleotide diversity among the analyzed non-coding regions of the *P1* alleles was calculated. A total of 19 indels and 30 polymorphic sites were found in the proximal promoter; while in the distal enhancer, a total of 1 indel and 17 polymorphic sites were identified. For the 1^st^*P1* intron, no indels were found, and a total of 6 polymorphic sites were detected (Additional file 1: Figure S1C), for this region the analysis of diversity denotes a high level of conservation (π-value 0.01667). We calculated two estimates of diversity from our data. Nucleotide polymorphism (θ) is calculated from the total number of segregating sites with correction to the sample size [32], and nucleotide diversity (π) is the probability that two randomly selected sequences will possess different nucleotides at a site [33]. The nucleotide polymorphism (θ) and diversity (π) estimated indicated that the distal enhancer region is more diverse than the proximal promoter region (Additional file 2: Table S2). This suggests that the distal enhancer may contain critical regulatory regions for *P1* expression. The analysis of variability can be correlated with the analysis performed for both phylograms on the non-coding regions of *P1*.

The radial trees show the diversity present between the landraces and the inbred lines for the proximal and distal promoter regions (Figure 4A). Evidently this region, near to the SST, may have found a greater selective pressure than the rest of the promoter during selection for better environmental traits under higher radiation levels found at high altitudes or even during domestication to obtain greater yield.

### Small RNAs expressed are complementary to a discrete region of the *P1* proximal enhancer

**Figure 5:**
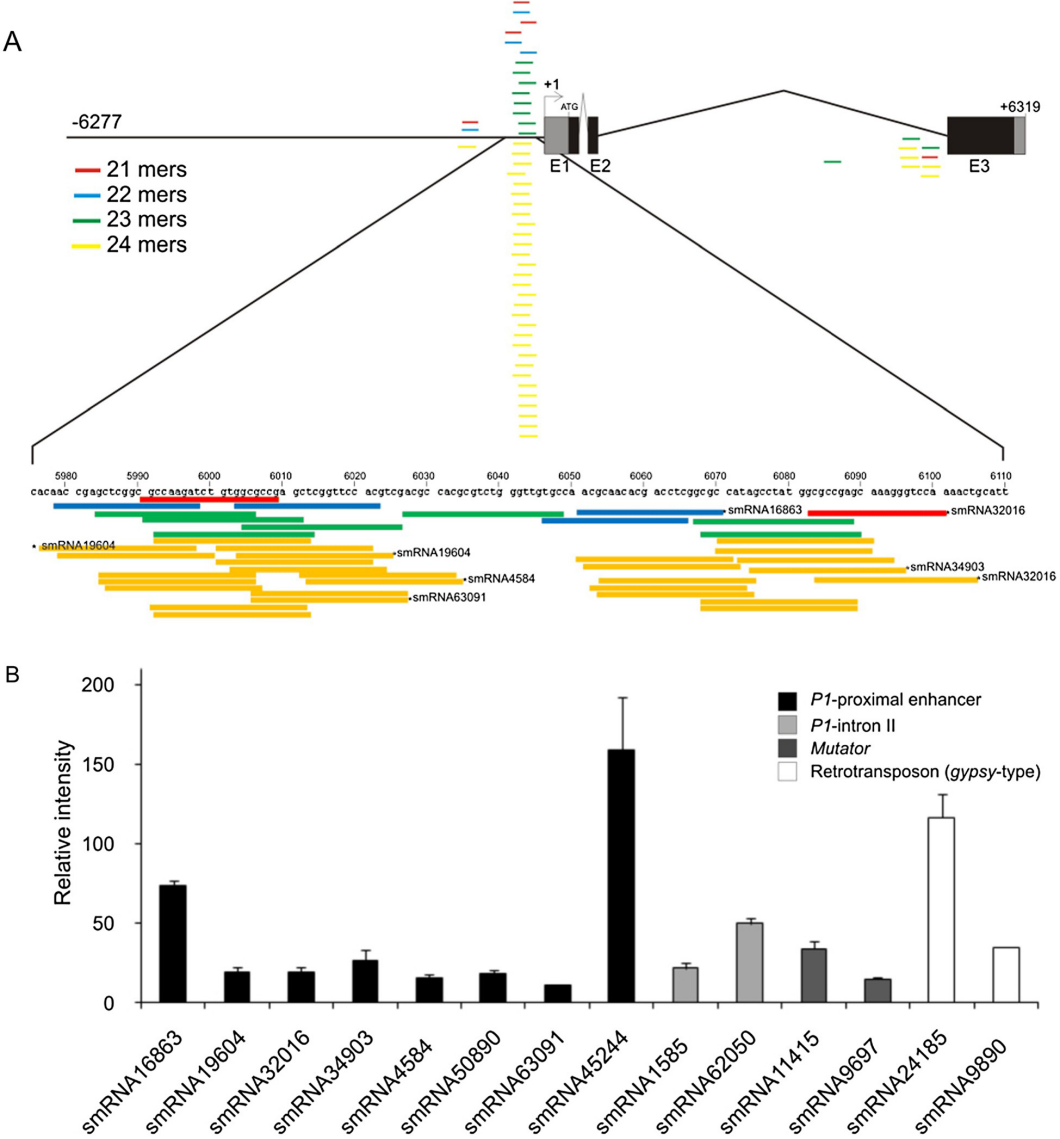
**siRNAs complementary to the*P1*sequence.** (**A**) Scheme of siRNAs complementary to the *P1* sequence (*P1*-siRNAs) obtained from the cereal small RNA library ( http://sundarlab.ucdavis.edu/smrnas/). A single copy of *P1*from B73 is represented. The *P1*-proximal enhancer is expanded to show the location of the *P1*-siRNAs identified. The siRNAs are shown as bars. Different colors represent different siRNA sizes, as indicated in the key. Asterisks show those siRNA assayed in the microarray experiment. (**B**) Validation of the data from the cereal small RNA library by microarray experiments using siRNAs from B73 leaves. Fifteen siRNAs were printed in the arrays as described in M&M. Only eight and two siRNAs corresponding to the proximal enhancer and 2^nd^ intron of *P1,* respectively, showed hybridization values at least three times higher than the background. *Mutator* and Retrotransposon (*gypsy*-type) siRNAs were used as positive controls [37].

The multicopy *P1* allele is epigenetically regulated, resulting in the phenotype *P1-wr* (white pericarp/red cob) due to hypermethylation [34,35]. To investigate if the low expression of *P1* in leaves of the B73 line is correlated with the presence of smRNAs complementary to different *P1* regions, smRNAs levels were measured by microarray analysis. Maize siRNA sequences were retrieved from the Cereal Small RNAs Database ( http://sundarlab.ucdavis.edu/smrnas/). Figure 5A shows all the siRNAs that are complementary to the genomic sequence of *P1*. The population includes all the species of 21 to 24 mers siRNAs, showing major proportion of 24mers. The highest density of siRNAs is localized on the proximal promoter (from −163 to −298) (zoomed in Figure 5B). Within this region of ~110 bps, a total of 41 complementary siRNAs were identified. We were also able to detect the presence of eight siRNA towards the 3’ end of the 2^nd^ intron. 

Using probes complementary to fifteen smRNA sequences, a microfluidic microarray-based assay was performed as described in Methods. After hybridizing with small RNAs extracted from B73 maize leaves, ten mature siRNAs showed significant expression levels (intensity >3fold background levels). However, siRNAs were detected at different intensity levels, ranging from 158.9 ± 33.2 to 11.2 ± 0.5 (Figure 5B). In addition, we were able to detect two smRNA which bind to 2^nd^ intron. This 2^nd^ intron region has also been demonstrated to be subject to epigenetic regulation [36]. The levels of expression observed for the smRNAs are in the same order of magnitude as those complementary to the *Mutator* transposon and *gypsy*-type retrotransposon (Figure 5B) [37,38]. It is interesting to note that the region of higher density of siRNAs is highly correlated with a zone of palindromes and repeated/inverted sequence within the *P1* gene (Additional file 3: Figure S2). Increased DNA and histone methylation are proposed to be triggered by double-stranded RNA, which is processed into 21–25 nucleotide RNA molecules [39]. Taking this into account, this short region of the proximal promoter could also be a fine regulation point of the expression of *P1* through a siRNA/methylation manner dependent.

### Mishca protoplasts activate *P1* expression in response to UV-B

The different *P1* expression pattern in the high altitude landraces can be attributed to the presence of *cis*- and/or *trans*- acting elements in the landraces, which may be absent or inactive in the inbred lines like B73 or W23. To address this point, *P1* promoter constructions using the *P1* basal promoter region (~1.1-kbp upstream from +1, Figure 4A) from Mishca and W23 were fused to the luciferase reporter gene, and these constructs were then used to transform Mishca, W23 and B73 protoplasts. These protoplasts were irradiated during 8 h with UV-B after transformation with p*P1*^MIS^::LUC or p*P1*^W23^::LUC, or kept under control conditions in the absence of UV-B, and luciferase activity was analyzed. As a control, protoplasts were co-transformed using a plasmid expressing Renilla under the constitutive promoter CaMV35S promoter (p35S::Renilla).

**Figure 6:**
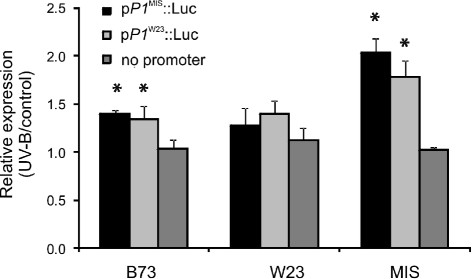
**Luciferase activity in transformed B73, W23 and Mishca protoplasts.** Protoplasts were isolated from etiolated maize leaves from B73, W23 and Mishca (MIS) genotypes. *P1* promoter fusions (~1.1kbp upstream +1) from W23 and Mishca were fused to the reporter gene Luciferase (p*P1*^MIS^::LUC and p*P1*^W23^::LUC, respectively), and protoplasts were transformed with the different constructs using Renilla luciferase vector as transformation control. Samples were then irradiated (UV- B) or not (control) during 8 h, and the luminescence of each sample was determined. The means of the results obtained from biological triplicates are shown; the error bars indicate the SDs of the samples. Asterisks denote statistical differences applying Student’s t test (*P* < 0.05).

When W23 transformed protoplasts that were irradiated during 8 h were analyzed, we did not observe any significant change in the LUC activity after the UV-B treatment, with neither of the two constructs (Figure 6). However, when B73 transformed protoplasts were used, the relative luminescence values (UVB/control) obtained showed a low, although significant and similar increase of about 30% in the luciferase activity for both constructs. Interestingly, the use of Mishca transformed protoplasts produced an even higher and similar increase (2.03-fold for the Mishca and 1.78 for the W23 promoter) than when B73 transformed protoplasts were used with the same two constructs after irradiation (Figure 6). Based on these results, and taking into account the high sequence identity between the Mishca and W23 promoter regions used, we hypothesize that the increase in the activity of the promoter using the Mishca protoplasts can be attributed to the presence of a P1 *trans*-regulatory factor active in Mishca cells in response to UV-B.

## Discussion

P1 is one of the transcription factors that control the flavonoid biosynthesis pathway in maize. While the mechanisms by which P1 controls gene expression have been well established ( [13,40]), little is known on how the expression of P1 is controlled. DNA methylation is clearly at play, evidenced by the distinct phenotypes of *P1-wr* and *P1-rr* ears [35,36]. In addition, the *Unstable Factor Orange1* (*UFO1*) allele plays a role controlling whether P1 is expressed in vegetative tissues or not [35,41-43]. The number of *P1* copies varies depending on the maize genotype. The *P1-rr* allele has been well characterized at the molecular level [44,45] and contains a single coding sequence, which when expressed, confers pigmentation to both the kernel pericarp and the cob [46]. Other allelic variants contain different *P1* copy numbers within their genomes. For example, the *P1-wr* allele is composed of six gene copies arranged in a tandem head-to-tail array ( [47]), and the B73 inbred line present a multiple copy *P1* cluster [25].

In addition, the multicopy *P1* allele is epigenetically regulated, resulting in the phenotype *P1-wr* (white pericarp/red cob) due to hypermethylation [34,35]. However, the degree of methylation is modulated by the presence of *Unstable factor for orange1* (*Ufo1*), which leads to ectopic pigmentation of pericarp and other organs when induces progressive hypomethylation over generations [43]. Paramutation was first described for the maize *Red1* (*R1*) gene [48]. Subsequently, three more regulatory genes of the flavonoid biosynthetic pathway, *B1* (*Booster1*), *PL1* (*Plant color1*), and *P1*[26,49,50] were shown to undergo paramutation in maize [63]. For example, the *P1-wr* gene copies are hypermethylated in their coding and non-coding regions relative to the *P1-rr* allele; the *P1-wr* multi-copy structure possibly results in intra-allelic interactions that give rise to silencing of *P1-wr* expression in the kernel pericarp [35]. Functional analysis of *P1-wr* promoter and coding sequences in transgenic maize plants, as well as studies of natural *P1* variants, have provided further support for the hypothesis that the organ-specific expression pattern of *P1-wr* is epigenetically regulated [41].

Here, we focused on the analysis of *P1* expression in leaves and its regulation by UV-B radiation in high altitude maize landraces, and its comparison with low altitude inbred lines, like B73 and W23. Using young leaves of five different maize high-altitude landraces, we found that P1 is induced by UV-B, both at the transcript and at the protein levels, in contrast to B73 or W23, which showed almost undetectable expression levels, and very low induction by UV-B. In addition, P1 is able to increase the transcription of the *CHS* gene after a UV-B treatment in Confite, while up-regulation of this gene by UV-B is not observed in W23 (Figure 2). Previously, we found that an 8 h-long UV-B-treatment can induce the expression of enzymes of the flavonoid pathway in maize [21]. Chalcone synthase (CHS) is a key enzyme in flavonoid biosynthesis. Maize has two CHS genes, C2 and WHP [22]. It had been shown that the expression of the *C2* gene is induced by low levels of UV-B/UV-A/blue light, and its function is largely regulated at the level of transcription [51-53]. In addition, ectopic expression of *P1* in maize protoplasts is also sufficient to induce *C2 in vivo*[14]. Thus, it is clear that P1 participates in the UV-B induction of the flavonoid pathway in leaves of high altitude landraces, but not in leaves of low altitude lines.

Taking into account the high allelic variability already described for *P1*[25,44,45,47], ranging from the simple-copy *P1-rr* allele, to the multiple-copy tandem organization present in the B73 genome, we estimated the copy number of *P1* in each genome of the high altitude landraces. Besides the high divergence that we found exists in the number of alleles present between the landraces (Figure 3), there is a correlation between the *P1* copy number and the place of origin of the different races. For instance, Arrocillo, Cacahuacintle and Conico, with the lowest copy number (1 to 3), were collected in Mexico, from altitudes ranging between 2200 and 2800 m.; while both the Andean South American races Mishca, which grows at 2200–2800 m, and Confite Puneño, which has adapted to live at 3600–3900 m, have 4 and 8 copies of *P1*, respectively. Evidently, this variability could be related to the selective pressure during the domestication to select adaptive traits, in this case to be able to grow under conditions of high incidence of UV-B.

We also cloned and sequenced two fragments of the *P1* promoter region (a distal and a proximal segment) and the first intron from the landraces under study, and from B73 and W23. The analysis allowed us to determinate the presence of different allelic variants, the presence of variable numbers of indels and the location of them within these promoter sequences. We also analyzed the presence of different DNA motifs that may participate in the regulation by UV-B in the promoter regions. For example, both the Mishca and the W23 *P1* promoter regions contain seven UVBoxes (CAAG) distributed within the ~1.1-kbp proximal promoter analyzed and one ACE^*CHS*^core (CACGT) [28] (Additional file 1: Figure S1). Assuming that this element is of importance in the induction by UV-B, we found only one difference between both promoter regions that could give different UV-B regulation, which is the unequal number of core ACE^*ANAC13*^ (ACGTG) located near the TSS, Mishca having three and only two present in W23. Another difference is the presence of a 24 bps sequence in the W23 promoter, which includes a sequence highly similar to a SIR (sub terminal inverted repeat, GCTCGGCGCCATAG) described by Goettel and Messing [25] of 14 bp that is also present the terminal inverted repeats of *Mutator*-like transposons. This indel produces a displacement of the ACE^*CHS*^ and all the UVBoxes from the ACE^*ANAC13*^ core in W23. However, when transient expression analysis using leaf protoplasts from W23 and Mishca, and proximal promoter constructs fused to the luciferase gene reporter from both lines were done (Figure 6), we found that differences in gene regulation by UV-B are largely a consequence of effects in *trans-*, suggesting that these regulatory sequence differences are not the causal reasons for the difference in expression. Rather, our results suggest the presence of a regulatory protein in Mishca protoplasts that responds to UV-B, which may be able to bind to regulatory sequences in the promoter of *P1*.

To further analyze other possible mechanisms that may participate in the regulation of the expression of *P1* in maize leaves, an analysis of the presence of small RNAs in leaves of the B73 line was conducted. Our results indicate that there is a high number of smRNAs complementary to a specific region in the basal *P1* promoter of about 100 bps, which includes a repeated-inverted sequence. This region is a target of more than 40 smRNAs (from 21 to 24 bps in length). Until now, little evidence existed respect to the mechanism and participation of these small RNAs in *P1* silencing. Analysis of these smRNAs levels in leaves of high altitude landraces, and their levels after UV-B exposure are currently under study to understand their possible involvement in the induction of *P1.*

## Conclusion

We have demonstrated that *P1* expression is increased in response to UV-B in high altitude maize landraces, and P1 binding to the *C2* promoter is increased by UV-B in at least one if these landraces. Although polymorphisms exist between the *P1* basal promoter sequences of the landraces and low altitude lines, we found that differences in gene regulation by UV-B are largely a consequence of effects in *trans-*, suggesting that these regulatory sequence differences are not the causal reasons for the difference in expression. The analysis of the presence of small RNAs in leaves of the B73 line show that there is a high number of smRNAs complementary to a specific region in the basal *P1* promoter, the role of these smRNAs in the regulation of *P1* leaf expression and UV-B regulation is under study*.*

## Methods

### Plant material and radiation treatments

Two *Zea mays* inbred lines, B73 and W23^*b,pl*^, and five high-altitude landraces (Arrocillo Amarillo (ARR), Cacahuacintle (CCH), Conico Norteño (CNC), from altitudes between 2200 and 2800 m., Mexico), and Mishca (MIS, from altitudes between 2200 and 2800 m.) and Confite Puneño (CFT, from altitudes between 3600 and 3900 m.a.s.l.), native from the South American Andes were used. The highland lines where obtained from the Maize Genetics Cooperation Stock Center, http://maizecoop.cropsci.uiuc.edu/USDA/ARS, University of Illinois, Urbana/Champaign National Plant Germplasm System (NPGS) and from International Maize and Wheat Improvement Center (CIMMYT), D.F. Mexico, Mexico. B73 seeds were obtained from the Instituto Nacional de Tecnología Agropecuaria (INTA, http://www.inta.gov.ar/) and the W23^*b,pl*^ maize line is a lab stock as previously described [21]. The landraces *P1* phenotype alleles are summarized in Table S2.

Kernels from the different ears were selected and grown in the greenhouse with supplemental visible light to 10% of summer noon radiation with approximately 15 h light/9 h dark without UV-B for 28 d. UV-B was provided once for 8 h, starting 3 h after the beginning of the light period, using fixtures mounted 30 cm above the plants (Phillips, F40UVB 40 W and TL 20 W/12) at a UV-B intensity of 2 W m^-2^, UV-A: 0.65 W m^-2^. The bulbs were covered with cellulose acetate to exclude wavelengths <280 nm. As a control, plants were exposed for 8 h under the same lamps covered with polyester film (no UV-B treatment, UV-B: 0.04 W m^-2^, UV-A: 0.4 W m^-2^). Lamp output was recorded using a UV-B/UV-A radiometer (UV203 A + B radiometer, Macam Photometrics, Ltd, Livingston, UK) to ensure that both the bulbs and filters provided the designated UV dosage in all treatments. The UV-B treatment experiments were repeated at least three times, and biological replicate leaf samples were collected immediately after each experiment.

### RNA isolation and reverse transcription reaction

RNA samples were isolated using Trizol (Invitrogen, Carlsbad, CA) as described by Casati and Walbot [21]. RNA was isolated from a pool of young leaves from 6 plants; pooling minimizes organismal variation. Five μg of total RNA from each genotype/treatment combination was used for cDNA synthesis using Superscript II reverse transcriptase (Invitrogen). Three biological replicates were used for each sample plus a negative control (reaction without reverse transcriptase).

For *P1* expression analyses of inbred and landraces lines under control and UV- B conditions, nested RT-PCR was performed as previously described [54]. For the first round PCR reaction, EP5-8 and P1-23568 primers were used [54]. A 5-μl portion of cDNA was added, and amplification conditions were as follows: 2 min denaturation at 94°C; 40 cycles at 94°C for 15 s, 60°C for 1 min and 72°C for 1 min, followed by 5 min at 72°C. For the second round PCR reaction, EP5-8-1 and ZFRT-8 primers were used [54]. After first-round PCR, the reaction product was diluted 1:10 with dH_2_O. The diluted samples were used for the second-round (nested) PCR. A 0.1 μl of the PCR product from the first PCR reaction was added, and amplification conditions were as follows: 2 min denaturation at 94°C; 40 cycles at 94°C for 15 sec, 50°C for 1 min and 72°C for 1 min, followed by 5 min at 72°C. Primers for a non-UV-B regulated gene, encoding a putative thioredoxin-*like* protein were used as a control [55].

### Quantitative PCR

Quantitative real-time PCR (qRT-PCR) was carried out in a MiniOPTICON2 (Bio-Rad, Richmond CA) as described in Casati and Walbot [55]. For qRT-PCR, three biological replicates were performed for each sample plus template-free samples and other negative controls (reaction without reverse transcriptase). Amplification of a thioredoxin-*like* transcript was used for data normalization. To confirm the size of the PCR products and to check for correspondence to a unique and expected PCR product, the final PCR products were analyzed on a 2% agarose gel.

### Antibodies and western blot

The P1 polyclonal antibodies (#344) generated against the C-terminal region of P1, which has no homology with other maize proteins (excluding the MYB domain), were used. The P1 polyclonal antibodies (#344) were generated against 125aa corresponding to the C-terminal region of P1 (excluding the MYB domain), were expressed as aHis_6_-tagged fusion protein in *E. coli* followed by affinity purification. For immunodetection, maize leaf nuclear proteins were obtained from nuclei enrichment extracts according to the procedure described by Casati et al. [56]. Nuclear proteins extracts from control and UV-B treated leaf samples were resolved on a 15% SDS-PAGE, transferred to a nitrocellulose membrane and probed with antibodies against P1 [57]. Thirty micrograms of nuclear proteins were loaded in each lane. Densitometric analysis of the gels and blots was performed using the Gel Pro Analyzer program (Media Cybernetics, Silver Spring, MD, USA).

### Promoter cloning

Total genomic DNA was extracted from leaf tissue as previously described [58]. To amplify the fragments corresponding to the basal promoter (~1.1 kb), distal promoter (~0.6 kb), and 1^st^ intron of *P1*, primers were designed based on the EF165349 sequence ( http://www.maizesequence.com). The fragments were amplified using the FailSafe™ PCR System Kit (Epicentre®) and cloned in the pGEMT-easy® (Promega) vector and sequenced. The basal promoter fragments were re-amplified by PCR using primers with the restriction sites *Not*I and *Kpn*I included in the forward and reverse primers, respectively (see Table S1). The PCR products were purified from gels, cut with the corresponding restriction enzymes and purified. The p*A1*::Luc construct (pMSZ011) [59] was restricted with *Not*I and *Kpn*I and the *A1* promoter was replaced by the *ZmP1* basal promoter (~1.1 kb), resulting in the p*P1*::Luc construct.

### Chromatin immunoprecipitation (ChIP) experiments

Mature leaves from CFT and W23 lines that were treated with UV-B and were kept under control conditions in the absence of UV-B were used for ChIP experiments. The antibody (#344) which specifically recognized the C-terminus of P1 was used for immunoprecipitation. Both PCR (25 cycles) and qPCR were carried out for the quantification of enrichment of P1 binding to sequences from −380 to −280 in the chalcone synthase promoter, primers used for the PCR analyses are shown in Table S1. Three biological replicates were performed from each genotype/treatment sample type, and three PCR experiments were done with each sample.

### smRNA microarray

The miRNA microarray was custom synthesized by LC Sciences (Houston, Texas, USA) using their protocols [60]. Briefly, poly-A tails were added to the 3’ ends of small RNAs using a poly(A) polymerase, and Cy3 and Cy5 was then ligated to the poly-A tails of samples from control and UV-B treated maize plants. The tagged RNAs were hybridized to the microfluidic hybridization chip. Hybridization images were scanned with a Gene- Pix 4000B microarray scanner (Molecular Devices, Sunnyvale, USA). Data extraction and image processing were performed using ArrayPro™ image analysis software (Media Cybernetics, Silver Spring, USA). A smRNA was scored as detectable if the signal intensity was higher than 3 times the standard deviation of the background signal, and the spot CV <0.5. CV is calculated as standard deviation/signal intensity; above background significant intensities had p-values < 0.05 using the SIGMA STAT package (SciencesSoftware.com, http://www.sciencessoftware.com). Normalization was performed with a LOWESS method to remove system-related variations.

### Maize protoplast transient assay for *P1* promoter activity

Protoplasts were isolated from etiolated maize seedlings (W23, B73 and Mishca) as described by Sheen [61]. Approximately 320.000 protoplasts were used in each of 200 μL reactions. For transient experiments, 20 μg of plasmid test DNA (p*P1*^MIS^::LUC or p*P1*^W23^::LUC) along with 5 μg renilla luciferase reporter plasmid were transformed into protoplasts. The reporter gene was driven by enhanced 35 S promoter and terminated by the 3’UTR of the nopaline synthase gene. Protoplasts without plasmid were used as controls. The protoplasts were transformed using a 40% PEG solution for 5 min at room temperature. Protoplasts were incubated for 8 h at 26°C under control and UV-B conditions in 16-well plates and then harvested by centrifugation (100 × *g* for 3 min). For the luciferase assays, 100 μL of the protoplast solution was removed to a 96-well assay plated and added 3 mL of 5X passive lysis buffer (Promega, Madison, WI, USA). The Dual-Luciferase Reporter assay kit from Promega was used to measure the renilla luciferase and firefly luciferase activities. The renilla luciferase and firefly luciferase activities were read on a LD-400 Luminescence detector (Beckman-Coulter) and the units were counts per second. To analyze the effect of the UV-B treatment on protoplast stability, protoplasts were transformed with a plasmid with the 35 S::GFP construct, and the integrity of the protoplasts was analyzed using an inverted confocal microscope Nikon C1/Eclipse TE-2000-E2 (see Additional file 4: Figure S3). Transient expression experiments in maize W23, MIS and B73 protoplast cells were performed with the p-*P1* (~1.1kpb)::LUC + p35S::Renilla, and p35S::Renilla plasmids. Transformation conditions of maize protoplast cells and transient expression assays for luciferase Firefly and Renilla were performed essentially as previously described [40,62].

### Accession numbers

Accession numbers for the nucleotide sequences of the different landraces promoters and distal enhancer of *P1* are deposited in GenBank®: proximal promoters: ARR (JX033024), CCH (JX033025), CFT-1 (JX033026), CFT-2(JX033027), MIS-1 (JX033028) and MIS-2 (JX033029). Distal enhancers: ARR (JX033030), CCH (JX033031), CFT (JX033032), CNC (JX033033) and MIS (JX033034).

### Statistical analysis

Data presented were analyzed using either the t-student test, or one-way analysis of variance (ANOVA). Minimum significant differences were calculated by the Bonferroni, Holm–Sidak, Dunett, and Duncan tests (*p* < 0.05) using the SIGMA STAT package (SciencesSoftware.com, http://www.sciencessoftware.com).

## Competing interests

The authors declare that they have no competing interests.

## Authors’ contributions

SPR, PC and EG conceived the experiments. SPR and PC did the experiments. The paper was written by SPR and PC, and edited by EG. All authors have read and approved the final manuscript.

## Supplementary Material

Additional file 1 **Table S1.** Sequence alignments corresponding to different P1 regions from high- altitude landraces and inbred lines. (A) Proximal promoter (~1100-bp), (B) distal enhancer (~600-bp) and (C) 1^st^ intron of *P1* gene alignments sequences. The location of primers used to detect copy number is highlighted in yellow in the distal enhancer (B). Click here for file

Additional file 2 **Table S1.** List of primers sequences. **Table S2.** Nucleotide diversity in the p1 alleles. Click here for file

Additional file 3 **Figure S2.** Dot matrix plot representing direct and inverted repeats of the sequence (108 bps) corresponding to the expanded region of the proximal promoter described in Figure 5A. Numbers indicate position relative to SST of *P1* on *x* and *y* axis. Click here for file

Additional file 4 **Figure S3.** Integrity of protoplasts irradiated with UV-B after 8 h of exposition. Left panel: protoplast not expressing GFP (control), right panel: protoplasts transformed with GFP. Click here for file

## References

[B1] Rizzini L Favory J Cloix C Faggionato D O'Hara A Kaiserli E Baumeister R Schafer E Nagy F Jenkins G Ulm R Perception of UV‒B by the Arabidopsis UVR8 protein Science 2011 332 103 106 10.1126/science.1200660 21454788

[B2] Britt AB DNA Damage and Repair in Plants Annu 1996 47 75 100 10.1146/annurev.arplant.47.1.75 15012283

[B3] Casati P Walbot V Crosslinking of ribosomal proteins to RNA in maize ribosomes by UV‒B and its effects on translation Plant Physiol 2004 136 3319 3332 10.1104/pp.104.047043 15466230 PMC523391

[B4] Gerhardt KE Wilson MI Greenberg BM Tryptophan photolysis leads to a UVB‒induced 66 kDa photoproduct of ribulose‒1,5‒bisphosphate carboxylase/oxygenase (Rubisco) in vitro and in vivo Photochem 1999 70 49 56

[B5] Bergo E Segalla A Giacometti GM Tarantino D Soave C Andreucci F Barbato R Role of visible light in the recovery of photosystem II structure and function from ultraviolet‒ B stress in higher plants J 2003 54 1665 1673 10.1093/jxb/erg180 12754266

[B6] Waterworth WM Jiang Q West CE Nikaido M Bray CM Characterization of Arabidopsis photolyase enzymes and analysis of their role in protection from ultraviolet‒B radiation J 2002 53 1005 1015 10.1093/jexbot/53.371.1005 11971912

[B7] Bieza K Lois R An Arabidopsis mutant tolerant to lethal ultraviolet‒B levels shows constitutively elevated accumulation of flavonoids and other phenolics Plant Physiol 2001 126 1105 1115 10.1104/pp.126.3.1105 11457961 PMC116467

[B8] Mazza CA Boccalandro HE Giordano CV Battista D Scopel AL Ballare CL Functional significance and induction by solar radiation of ultraviolet‒absorbing sunscreens in field-grown soybean crops Plant Physiol 2000 122 117 125 10.1104/pp.122.1.117 10631255 PMC58850

[B9] Madronich S Mckenzie RL Caldwell M Bjorn LO Changes in ultraviolet‒radiation reaching the earth’s surface AMBIO 1995 24 143 152 10.1016/s1011-1344(98)00182-1 9894350

[B10] Balis DS Zerefos CS Kourtidis K Bais AF Hofzumahaus A Kraus A Schmitt R Blumthaler M Gobbi GP Measurements and modeling of photolysis rates during the photochemical activity and ultraviolet radiation (PAUR) II campaign J 2002 107 1 12

[B11] Stapleton AE Walbot V Flavonoids can protect maize DNA from the induction of ultraviolet-radiation damage Plant Physiol 1994 105 881 889 10.1104/pp.105.3.881 8058838 PMC160736

[B12] Landry LC Chapple CCS Last R Arabidopsis mutants lacking phenolic sunscreens exhibit enhanced ultraviolet‒B injury and oxidative damage Plant Physiol 1995 109 1159 1166 10.1104/pp.109.4.1159 8539286 PMC157646

[B13] Grotewold E Drummond BJ Bowen B Peterson T The myb‒homologous P gene controls phlobaphene pigmentation in maize floral organs by directly activating a flavonoid biosynthetic gene subset Cell 1994 76 543 553 10.1016/0092-8674(94)90117-1 8313474

[B14] Grotewold E Chamberlin M Snook M Siame B Butler L Swenson J Maddock S St Clair G Bowen B Engineering secondary metabolism in maize cells by ectopic expression of transcription factors Plant Cell 1998 10 721 740 9596632 PMC144024

[B15] Quattrocchio F Baudry A Lepiniec L Grotewold E Grotewold E The regulation of flavonoid biosynthesis The science of flavonoids 2006 New York: Springer 97 122

[B16] Grotewold E The science of flavonoids 2006 New York: Springer

[B17] Casati P Walbot V Differential accumulation of maysin and rhamnosylisoorientin in leaves of high‒altitude landraces of maize after UV‒B exposure Plant Cell Environ 2005 28 788 799 10.1111/j.1365-3040.2005.01329.x

[B18] Gerdes JT Tracy WF Diversity of historically important sweet corn inbreds as estimated by RFLPs, morphology, isozymes, and pedigree Crop Sci 1994 34 26 33 10.2135/cropsci1994.0011183X003400010004x

[B19] Sheridan WF Maize for biological research 1982 Grand Forks, ND: Plant Molecular Biology Association, University Press

[B20] Falcone Ferreyra ML Rius SP Emiliani J Pourcel L Feller A Morohashi K Casati P Grotewold E Cloning and characterization of a UV‒B‒inducible maize flavonol synthase Plant J 2010 62 77 91 10.1111/j.1365-313X.2010.04133.x 20059741

[B21] Casati P Walbot V Gene expression profiling in response to ultraviolet radiation in maize genotypes with varying flavonoid content Plant Physiol 2003 132 1739 1754 10.1104/pp.103.022871 12913132 PMC181262

[B22] Franken P Niesbach‒Klosgen U Weydemann U Marechal‒Drouard L Saedler H Wienand U The duplicated chalcone synthase genes C2 and Whp (white pollen) of Zea mays are independently regulated; evidence for translational control of Whp expression by the anthocyanin intensifying gene in EMBO J 1991 10 2605 2612 1714383 10.1002/j.1460-2075.1991.tb07802.x PMC452959

[B23] Rudenko G Kurtz R Batey D Walbot V Determining Transgene Copy Number Using Real‒Time qPCR on the MJ Research® Opticon™ 2 Continuous Fluorescence Detection System Real‒Time Detection, Application Note 2004 Vol.2, No.11

[B24] Zhang P Wang Y Zhang J Maddock S Snook M Peterson T A maize QTL for silk maysin levels contains duplicated Myb‒homologous genes which jointly regulate flavone biosynthesis Plant Mol 2003 52 1 15 10.1023/A:1023942819106 12825685

[B25] Goettel W Messing J Change of gene structure and function by non‒homologous end‒joining, homologous recombination, and transposition of DNA PLoS Genet 2009 5 e1000516 10.1371/journal.pgen.1000516 19521498 PMC2686159

[B26] Sidorenko LV Peterson T Transgene‒induced silencing identifies sequences involved in the establishment of paramutation of the maize p1 gene Plant Cell 2001 13 319 335 11226188 10.1105/tpc.13.2.319 PMC102245

[B27] Hartmann U Valentine WJ Christie JM Hays J Jenkins GI Weisshaar B Identification of UV/blue light-response elements in the Arabidopsis thaliana chalcone synthase promoter using a homologous protoplast transient expression system Plant Mol 1998 36 741 754 10.1023/A:1005921914384 9526507

[B28] Safrany J Haasz V Mate Z Ciolfi A Feher B Oravecz A Stec A Dallmann G Morelli G Ulm R Nagy F Identification of a novel cis‒regulatory element for UV-B-induced transcription in Arabidopsis Plant J 2008 54 402 414 10.1111/j.1365-313X.2008.03435.x 18266923

[B29] Bhattramakki D Dolan M Hanafey M Wineland R Vaske D Register JC Tingey SV Rafalski A Insertion‒deletion polymorphisms in 3′ regions of maize genes occur frequently and can be used as highly informative genetic markers Plant Mol 2002 48 539 547 10.1023/A:1014841612043 12004893

[B30] Martin C Paz‒Ares J MYB transcription factors in plants Trends in Genetics 1997 13 67 73 10.1016/S0168-9525(96)10049-4 9055608

[B31] Llaca V Campbell MA Deschamps S Genome diversity in maize J. Botany 2011 2011

[B32] Watterson GA On the number of segregating sites in genetical models without recombination Theor 1975 7 256 276 10.1016/0040-5809(75)90020-9 1145509

[B33] Tenaillon MI Sawkins MC Long AD Gaut RL Doebley JF Gaut BS Patterns of DNA sequence polymorphism along chromosome 1 of maize (Zea mays ssp. mays L.) Proc 2001 98 9161 9166 10.1073/pnas.151244298 PMC55390 11470895

[B34] Chopra S Brendel V Zhang J Axtell JD Peterson T Molecular characterization of a mutable pigmentation phenotype and isolation of the first active transposable element from Sorghum bicolor Proc 1999 96 15330 15335 10.1073/pnas.96.26.15330 PMC24819 10611384

[B35] Chopra S Cocciolone SM Bushman S Sangar V McMullen MD Peterson T The maize Unstable factor for orange1 is a dominant epigenetic modifier of a tissue specifically silent allele of pericarp color1 Genetics 2003 163 1135 1146 12663550 10.1093/genetics/163.3.1135 PMC1462483

[B36] Sekhon RS Peterson T Chopra S Epigenetic modifications of distinct sequences of the p1 regulatory gene Sspecify tissue-specific expression patterns in maize Genetics 2007 175 1059 1070 17179091 10.1534/genetics.106.066134 PMC1840062

[B37] Questa JI Walbot V Casati P Mutator transposon activation after UV‒B involves chromatin remodeling Epigenetics 2010 5 352 363 10.4161/epi.5.4.11751 20421734 PMC2911508

[B38] Wang X Elling AA Li X Li N Peng Z He G Sun H Qi Y Liu XS Deng XW Genome‒wide and organ‒specific landscapes of epigenetic modifications and their relationships to mRNA and small RNA transcriptomes in maize Plant Cell 2009 21 1053 1069 10.1105/tpc.109.065714 19376930 PMC2685623

[B39] Li B Carey M Workman JL The role of chromatin during transcription Cell 2007 128 707 719 10.1016/j.cell.2007.01.015 17320508

[B40] Hernandez JM Feller A Morohashi K Frame K Grotewold E The basic helix loop helix domain of maize R links transcriptional regulation and histone modifications by recruitment of an EMSY‒related factor Proc. Natl. Acad. Sci. U S A 2007 104 17222 17227 10.1073/pnas.0705629104 17940002 PMC2040437

[B41] Cocciolone SM Chopra S Flint‒Garcia SA McMullen MD Peterson T Tissue‒specific patterns of a maize Myb transcription factor are epigenetically regulated Plant J 2001 27 467 478 10.1046/j.1365-313X.2001.01124.x 11576430

[B42] Cocciolone SM Sidorenko LV Chopra S Dixon PM Peterson T Hierarchical patterns of transgene expression indicate involvement of developmental mechanisms in the regulation of the maize P1‒rr promoter Genetics 2000 156 839 846 11014829 10.1093/genetics/156.2.839 PMC1461292

[B43] Sekhon RS Chopra S Progressive loss of DNA methylation releases epigenetic gene silencing from a tandemly repeated maize Myb gene Genetics 2009 181 81 91 19001287 10.1534/genetics.108.097170 PMC2621191

[B44] Athma P Grotewold E Peterson T Insertional mutagenesis of the maize P gene by intragenic transposition of Ac Genetics 1992 131 199 209 1317315 10.1093/genetics/131.1.199 PMC1204954

[B45] Lechelt C Peterson T Laird A Chen J Dellaporta SL Dennis E Peacock WJ Starlinger P Isolation and molecular analysis of the maize P locus Mol 1989 219 225 234 10.1007/BF00261181 2559311

[B46] Grotewold E Athma P Peterson T Alternatively spliced products of the maize P gene encode proteins with homology to the DNA‒binding domain of myb‒like transcription factors Proc. Natl. Acad. Sci. U S A 1991 88 4587 4591 10.1073/pnas.88.11.4587 2052542 PMC51710

[B47] Chopra S Athma P Li X Peterson T A maize Myb homolog is encoded by a multicopy gene complex Mol Gen Genet 1998 260 372 380 10.1007/s004380050906 9870702

[B48] Brink RA A genetic change associated with the R locus in maize which is directed and potentially reversible Genetics 1956 41 872 889 17247669 10.1093/genetics/41.6.872 PMC1224369

[B49] Coe EH A regular and continuing conversion‒type phenomenon at the B locus in maize Proc. Natl. Acad. Sci. U S A 1959 45 828 832 10.1073/pnas.45.6.828 16590451 PMC222644

[B50] Hollick JB Patterson GI Coe EHJ Cone KC Chandler VL Allelic interactions heritably alter the activity of a metastable maize pl allele Genetics 1995 141 709 719 8647404 10.1093/genetics/141.2.709 PMC1206767

[B51] Feinbaum RL Storz G Ausubel FM High intensity and blue light regulated expression of chimeric chalcone synthase genes in transgenic Arabidopsis thaliana plants Mol Gen 1991 226 449 456 10.1007/BF00260658 2038307

[B52] Jenkins GI Long JC Wade HK Shenton MR Bibikova TN UV and blue light signalling: pathways regulating chalcone synthase gene expression in Arabidopsis New Phytol 2001 151 121 131 10.1046/j.1469-8137.2001.00151.x 33873370

[B53] Taylor LP Briggs WR Genetic regulation and photocontrol of anthocyanin Accumulation in maize seedlings Plant Cell 1990 2 115 127 2136630 10.1105/tpc.2.2.115 PMC159869

[B54] Zhang F Comparisons of maize pericarp color1 alleles reveal paralogous gene recombination and an organ-specific enhancer region Plant Cell 2005 17 903 914 10.1105/tpc.104.029660 15722466 PMC1069707

[B55] Casati P Walbot V Rapid transcriptome responses of maize (Zea mays) to UV‒B in irradiated and shielded tissues Genome Biol 2004 5 R16 10.1186/gb-2004-5-3-r16 15003119 PMC395766

[B56] Casati P Campi M Chu F Suzuki N Maltby D Guan S Burlingame AL Walbot V Histone acetylation and chromatin remodeling are required for UV‒B‒dependent transcriptional activation of regulated genes in maize Plant Cell 2008 20 827 842 10.1105/tpc.107.056457 18398050 PMC2390752

[B57] Burnette WN "Western blotting": electrophoretic transfer of proteins from sodium dodecyl sulfate‒polyacrylamide gels to unmodified nitrocellulose and radiographic detection with antibody and radioiodinated protein A Anal 1981 112 195 203 10.1016/0003-2697(81)90281-5 6266278

[B58] Nan G‒L Walbot V Non-radioactive genomic DNA blots for detection of low abundant sequences in transgenic maize Methods Mol Biol 2009 113 122 19377998 10.1007/978-1-59745-494-0_9

[B59] Sainz MB Grotewold E Chandler VL Evidence for direct activation of an anthocyanin promoter by the maize C1 protein and comparison of DNA binding by related Myb domain proteins Plant Cell 1997 9 611 625 9144964 10.1105/tpc.9.4.611 PMC156943

[B60] Gao X Gulari E Zhou X In situ synthesis of oligonucleotide microarrays Biopolymers 2004 73 579 596 10.1002/bip.20005 15048782

[B61] Sheen J A transient expression assay using Arabidopsis mesophyll protoplasts 2002 http://genetics.mgh.harvard.edu/sheenweb/

[B62] Feller A Hernandez JM Grotewold E An ACT-like domain participates in the dimerization of several plant basic-helix-loop-helix transcription factors J 2006 281 28964 28974 10.1074/jbc.M603262200 16867983

[B63] Arteaga‒Vazquez MA Chandler VL Paramutation in maize: RNA mediated trans‒generational gene silencing Curr 2010 20 156 163 10.1016/j.gde.2010.01.008 PMC2859986 20153628

