# Supplementary material for: Analysis of the P1 promoter in response to UV-B radiation in allelic variants of high-altitude maize
Source: BMC Plant Biol. 2012 Jun 15;12:92. doi: 10.1186/1471-2229-12-92 (PMC3489873; doi:10.1186/1471-2229-12-92)
Supplement: Additional file 1 — Table S1. Sequence alignments corresponding to different P1 regions from high- altitude landraces and inbred lines. (A) Proximal promoter (~1100-bp), (B) distal enhancer (~600-bp) and (C) 1st intron of P1 gene alignments sequences. The location of primers used to detect copy number is highlighted in yellow in the distal enhancer (B). [file 1471-2229-12-92-S1.pdf]

Figure S1

A

Proximal enhancer (~1.1-kbp)

```
                                        *
B73      CATATATCGGCCTGGGATTGCAAGCGAGCAGGCAACGCTGTGCGGGAGTGCGGCCTGCGG 60
W23      CATATATCGGCCTGGGATTGCAAGCGAGCAGGCAACGCTGTGCGGGAGTGCGGCCTGC-- 58
ARR      CATATATCGGCCTGGGATTGCAAGCGAGCAGGCAACGCTGTGCGGGAGTGCGGCCTGC-- 58
CCH      CATATATCGGCCTGGGATTGCAAGCGAGCAGGCAACGCTGTGCGGGAGTGCGGCCTGCGG 60
CFT-1    CATATATCGGCCTGGGATTGCAAGCGAGCAGGCAACGCTGTGCGGGAGTGCGGCCTGC-- 58
CFT-2    CATATATCGGCCTGGGATTGCAAGCGAGCAGGCAACGCTGTGCGGGAGTGCGGCCTGC-- 58
MIS-1    CATATATCGGCCTGGGATTGCAAGCGAGCAGGCAACGCTGTGCGGGAGTGCGGCCTGCGG 60
MIS-2    CATATATCGGCCTGGGATTGCAAGCGAGCAGGCAACGCTGTGCGGGAGTGCGGCCTGCGG 60
         **********************************************************

           15-nt
B73      GAGTGCGGCCTGCTCGGTTGTGTTATTAAAATATTTGTTGCAGACATGAGCACAAAGCTC 120
W23      -------------TCGGTTGTGTTATTAAAATATTTGTTGCAGACATGAGCACAAAGCTC 105
ARR      -------------TCGGTTGTGTTATTAAAATATTTGTTGCAGACATGAGCACAAAGCTC 105
CCH      GAGTGCGGCCTGCTCGGTTGTGTTATTAAAATATTTGTTGCAGACATGAGCACAAAGCTC 120
CFT-1    -------------TCGGTTGTGTTATTAAAATATTTGTTGCAGACATGAGCACAAAGCTC 105
CFT-2    -------------TCGGTTGTGTTATTAAAATATTTGTTGCAGACATGAGCACAAAGCTC 105
MIS-1    GAGTGCGGCCTGCTCGGTTGTGTTATTAAAATATTTGTTGCAGACATGAGCACAAAGCTC 120
MIS-2    GAGTGCGGCCTGCTCGGTTGTGTTATTAAAATATTTGTTGCAGACATGAGCACAAAGCTC 120
                      ***********************************************

                                  *                            *
B73      ATCTAGCCCACTTGGTAGAGCACAAGGCTTCTAACCATGTGGTCGTGGGTTCAAGCCCCA 180
W23      ATCTAGCCCACTTGGTAGAGCACAAGGCTTCTAACCATGTGGTCGTGGGTTCAAGCCCCA 165
ARR      ATCTAGCCCACTTGGTAGAGCACAAGGCTTCTAACCATGTGGTCGTGGGTTCAAGCCCCA 165
CCH      ATCTAGCCCACTTGGTAGAACACAAGGCTTCTAACCATGTGGTCGTGGGTTCCAGCCCCA 180
CFT-1    ATCTAGCCCACTTGGTAGAGCACAAGGCTTCTAACCATGTGGTCGTGGGTTCAAGCCCCA 165
CFT-2    ATCTAGCCCACTTGGTAGAGCACAAGGCTTCTAACCATGTGGTCGTGGGTTCAAGCCCCA 165
MIS-1    ATCTAGCCCACTTGGTAGAGCACAAGGCTTCTAACCATGTGGTCGTGGGTTCAAGCCCCA 180
MIS-2    ATCTAGCCCACTTGGTAGAGCACAAGGCTTCTAACCATGTGGTCGTGGGTTCAAGCCCCA 180
         ******************* ******************************** *******

                                                    *
B73      TAGTTTGCATTTTTTTT--GTTTTTTTGTTTATGTCGTGTGTTCAAGCCCCATAGTTCCG 238
W23      TAGTTTGCATTTTTTTT--GTTTTTTTGTTTATGTCGTGGGTTCAAGCCCCATAGTTCCG 223
ARR      TAGTTTGCATTTTTTTT--GTTTTTTTGTTTATGTCGTGGGTTCAAGCCCCATAGTTCCG 223
CCH      TAGTTTGCATTTTTTTT--GTTTTTTTGTTTATGTCGTGGGTTCAAGCCCCATAATTCCG 238
CFT-1    TAGTTTGCATTTTTTTT--GTTTTTTTGTTTATGTCGTGGGTTCAAGCCCCATAGTTCCG 223
CFT-2    TAGTTTGCATTTTTTTT--GTTTTTTTGTTTATGTCGTGGGTTCAAGCCCCATAGTTCCG 223
MIS-1    TAGTTTGCATTTTTTTTTTGTTTTTTTGTTTATGTCGTGGGTTCAAGCCCCATAGTTCCG 240
MIS-2    TAGTTTGCATTTTTTTTTTGTTTTTTTGTTTATGTCGTGGGTTCAAGCCCCATAGTTCCG 240
         *****************  ******************** ************** *****

B73      CTTAAATTTATTTCTCGCCTAGATTTTTTTT--CACAATTGAAAAAATCGACCCAAAATA 296
W23      CTTAAATTTATTTCTCGCCTAGATTTTTTTTTTCACAATTGAAAAAATCGACCCAAAATA 283
ARR      CTTAAATTTATTTCTCGCCTAGATTTTTTTTT-CACAATTGAAAAAATCGACCCAAAATA 282
CCH      CTTAAATTTATTTCT-GCCTACATTTTTTTTT-CACAATTGAAAAAATCGACCCAAAATA 296
CFT-1    CTTAAATTTATTTCTCGCCTAGATTTTTTTTT-CACAATTGAAAAAATCGACCCAAAATA 282
CFT-2    CTTAAATTTATTTCTCGCCTAGATTTTTTTTT-CACAATTGAAAAAATCGACCCAAAATA 282
MIS-1    CTTAAATTTATTTCTCGCCTAGATTTTTTTTT-CACAATTGAAAAAATCGACCCAAAATA 299
MIS-2    CTTAAATTTATTTCTCGCCTAGATTTTTTTTT-CACAATTGAAAAAATCGACCCAAAATA 299
         *************** ***** *********  ***************************

B73      TATGCTCATGTACTGATCGGCCAATATCTCTGTATGTGAAAGGTTGTGGAGAATAATAAT 356
W23      TATGCTCATGTACTGATCGGCCAATATCTCTGTATGTGAAAGGTTGTGGAGAATAATAAT 343
ARR      TATGCTCATGTACTGATCGGCCAATATCTCTGTATGTGAAAGGTTGTGGAGAATAATAAT 342
CCH      TATGCTCATGTACTGATCGGCCAATATCTCTGTATGTGAAAGGTTGTGGAGAATAATAAA 356
CFT-1    TATGCTCATGTACTGATCGGCCAATATCTCTGTATGTGAAAGGTTGTGGAGAATAATAAT 342
CFT-2    TATGCTCATGTACTGATCGGCCAATATCTCTGTATGTGAAAGGTTGTGGAGAATAATAAT 342
MIS-1    TATGCTCATGTACTGATCGGCCAATACCTCTGTATGTGAAAGGTTGTGGAGAATAATAAT 359
MIS-2    TATGCTCATGTACTGATCGGCCAATACCTCTGTATGTGAAAGGTTGTGGAGAATAATAAT 359
         ************************** ******************************** 
```

```
B73            AAGTAGGGCATGCTGTTTATCAAAGCAAATGTATATAAGGAAGAAAAAAATGTATAAAAA 416
W23            AAGTAGGGCATGCTGTTTATCAAAGCAAATGTATATAAGGAAGAAAAAAATGTATAAAAA 403
ARR            AAGTAGGGCATGCTGTTTATCAAAGCAAATGTATATAAGGAAGAAAAAAATGTATAAAAA 402
CCH            AAGCAGGGCATGCTGTTTATCAAAGCAA-TGTATATAAGGAAGAAAAAAATGTATAAAAA 415
CFT-1          AAGTAGGGCATGCTGTTTATCAAAGCAAATGTATATAAGGAAGAAAAAAATGTATAAAAC 402
CFT-2          AAGTAGGGCATGCTGTTTATCAAAGCAAATGTATATAAGGAAGAAAAAAATGTATAAAAC 402
MIS-1          AAGTAGGGCATGCTGTTTATCAAAGCAAATGTATATAAGGAAGAAAAAAATGTATAAAAA 419
MIS-2          AAGTAGGGCATGCTGTTTATCAAAGCAAATGTATATAAGGAAGAAAAAAATGTATAAAA- 418
               *** ************************ ******************************

B73            TATTTATAGTGATTTAGAAATAGTTAATGATTCGTAATGCAAATTTTGAATAATGCACGG 476
W23            TATTTATAGTGATTTAGAAATAGTTAATGATTCGTAATGCAAATTTTGAATAATGCACGG 463
ARR            TATTTATAGTGATTTAGAAATAGTTAATGATTCGTAATGCAAATTTTGAATAATGCACGG 462
CCH            TATTTATAGTGATTTAGAAATAGTTAATGATTCGTAATGCAAATTTTGAATAATGCACGG 475
CFT-1          TATTTATAGTGATTTAGAAATAGTTAATGATTCGTAATGCAAATTTTGAATAATGCACGG 462
CFT-2          TATTTATAGCGATTTAGAAATAGTTAATGATTCGTAATGCAAATTTTGAATAATGCACGG 462
MIS-1          TATTTATAGTGATTT-GAAATAGTTAATGATTCGTAATGCAAATTTTGAATAAAGCACGG 478
MIS-2          TATTTATAGTGATTT-GAAATAGTTAATGATTCGTAATGTAAATTTTGAATAAAGCACGG 477
               ********* ***** *********************** ************* ******

B73            ATGACATTTTATAAAATTACTACATTGCTTTTGTATTGCACATGCATGATTTGAGCTAGT 536
W23            ATGACATTTTATAAAATTACTACATTGCTTTTGTATTGCACATGCATGATTTGAGCTAGT 523
ARR            ATGACATTTTATAAAATTACTACATTGCTTTTGTATTGCACATGCATGATTTGAGCTAGT 522
CCH            ATGACATTTTATAAAATTACTACATTGCTTTTGTATTGCACATGCATGATTTGAGCTAGT 535
CFT-1          ATGACATTTTATAAAATTACTACATTGCTTTTGTATTGCACATGCATGATATGAGCTAGT 522
CFT-2          ATGACATTTTATAAAATTACTACATTGCTTTTGTATTGCACATGCATGATTTGAGCTAGT 522
MIS-1          ATGACATTTTATAAAATTACTACATTGCTTTTGTATTGCACATGCATGATTTGAGCTAGT 538
MIS-2          ATGACATTTTATAAAATTACTACATTGCTTTTGTATTGCACATGCATGATTTGAGCTAGT 537
               ************************************************** *********

B73            CGATTATTTACGCGCATTTTAAATTCGGAAACTGTAGATTGAAATGCGCGCGCATGCAGT 596
W23            CGATTATTTACGCGCATTTTAAATTCGGAAACTGTAGATTGAAATGCGCGCGCATGCAGT 583
ARR            CGATTATTTACGCGCATTTTAAATTCGGAGACTGTAGATTGAAATGCGCGCGCATGCAGT 582
CCH            CGATTATTTACGCGCATTTTAAATTCGGAAACTGTAGATTGAAATGCGCGCGCATGCAGT 595
CFT-1          CGATTATTTACGCGCATTTTAAATTCGGAAACTGCAGATTGAAATGCACGCGAAAGCATT 582
CFT-2          CGATTATTTACGCGCATTTTAAATTCGGAAACTGTAGATTGAAATGCGCGCGCATGCAGT 582
MIS-1          CGATTATTTACGCGCATTTTAAATTCGGAAACTGTAGATTGAAATGCGCGCGCATGCAGT 598
MIS-2          CGATTATTTACGCGCATTTTAAATTCGGAAACTGTAGATTGAAATGCGCGCGCATGCAGT 597
               ***************************** **** ************ **** * *** *

                  *
B73            GCAAGTATGGAAGGCAACACTAGGCACAACGACATAAAAAAAA-TCTAGGCGAGAAATAA 655
W23            GCAAGTATGGAAGGCAACACTAGGCACAACGACATAAAAAAAA-TCTAGGCGAGAAATAA 642
ARR            GCAAGTATGGAAGGCAACACTAGGCACAACGACATAAAAAAAA-TCTAGGCGAGAAATAA 641
CCH            GCAAGTATGGAAGGCAACACTAGGCACAACGACATAAAAAAAA-TCTAGGCGAGAAATAA 654
CFT-1          GCTA-CATGGAAGGCAACACTAGGCACAACGACATAAAAAAAAATCTAGGCGAGAAATAA 641
CFT-2          GCAAGTATGGAAGGCAACACTAGGCACAACGACATAAAAAAAAATCTAGGCGAGAAATAA 642
MIS-1          GCAAGTATGGAAGGCAACACTAGGCACAACGACATAAAAAAAA-TCTAGGCGAGAAATAA 657
MIS-2          GCAAGTATGGAAGGCAACACTAGGCACAACGACATAAAAAAAA-TCTAGGCGAGAAATAA 656
               ** *  ************************************* ****************

B73            ATTTAAGCGGACACACCAACGACATAAACAAAAAAAAACAAAAAAAATGCAAACTATGGG 715
W23            ATTTAAGCGGACACACCAACGACATAAACAAAAAAAAACAAAAAAAATGCAAACTATGGG 702
ARR            ATTTAAGCGGACACACCAACGACATAAACAAAAAAAA-CAAAAAAAATGCAAACTATGGG 700
CCH            ATTTAAGCGGACACACCAACGACATAAACAAAAAAAAACAAAAAAAATGCAAACTATGGG 714
CFT-1          ATTTAAGCGGACACACCAACGACATAAACAAAAAAAAACAAAAAAAATGCAAACTATGGG 701
CFT-2          ATTTAAGCGGACACACCAACGACATAAACAAAAAAAAACAAAAAAAATGCAAACTATGGG 702
MIS-1          ATTTAAGCGGACACACCAACGACATAAACAAAAAAAAACAAAAAAAATGCAAACTATGGG 717
MIS-2          ATTTAAGCGGACACACCAACGACATAAACAAAAAAAAACAAAAAAAATGCAAACTATGGG 716
               ************************************* **********************
```

```
                                                   *
B73       GTTTGAACCC-ACGACCACATGGTT-AGAAG--CCTTGTGCTCTACCAAGTGGGCTAGAT 771
W23       GTTTGAACCC-ACGACCACATGGTT-AGAAG--CCTTGTGCTCTACCAAGTGGGCTAGAT 758
ARR       GTTTGAACCC-ACGACCACATGGTT-AGAAG--CCTTGTGCTCTACCAAGTGGGCTAGAT 756
CCH       GTTTGAACCC-ACAACCACATGGTT-AGAAG--CCTTGTGCTCTACCAAGTGGGCTAGAT 770
CFT-1     GTTTGAACCC-ACGACCACATGGTTTAGAAAGCCCTTGTGCTCTACCAAGTGGGCTAGAT 760
CFT-2     GTTTGAACCC-ACGACCACATGGTTTAGAAAGCCCTTGTGCTCTACCAAGTGGGCTAGAT 761
MIS-1     GTTTGAACCCCACGACCACATGGCT-AGAAG--CCTTGTGCTCTACCAAGTGGGCTAGAT 774
MIS-2     GTTTGAACCCCACGACCACATGGCT-AGAAG--CCTTGTGCTCTACCAAGTGGGCTAGAT 773
          ********** ** ********* * ****   ***************************

                                                               *
W23       GGGCTTTGTGCTCATGTCTGCAACAAATATTTTAATAACACAACCGAGCTCGGCGCCAAG 818
B73       GGGCTTTGTGCTCATGTCTGCAACAAATATTTTAATAACACAACCGAGCTCGGCGCCAAG 831
ARR       GGGCTTTGTGCTCATGTTTGCAACAAATATTTTAATAACACAACCGAGCTCGGCGCCAAG 816
CCH       GGGCTTTATGCTCATGTCTGCAACAAATATTTTAATAACACAACCGAGCTCGGCGCCAAG 830
CFT-1     GGGCTTTGTGCTCATGTT-GCAACAAATATTTTAATAACACAACCGAGCTCGGCGCCAAG 819
CFT-2     GGGCTTTGTGCTCATGTT-GCAACAAATATTTTAATAACACAACCGAGCTCGGCGCCAAG 820
MIS-1     GAGCTTTGTGCTCATGTCTGCCACAAATATTTTAATAACACAACCGAGCTCGGCGCCAAG 834
MIS-2     GAGCTTTGTGCTCATGTCTGCCACAAATATTTTAATAACACAACCGAGCTCGGCGCCAAG 833
          * ***** *********  ** ***************************************

                                  +++
W23       ATCTGTGGCGCCGAGCTCGGTTCCACGTCGACGCCACGCGTCTGGGTTGTGCCAACGCAA 878
B73       ATCTGTGGCGCCGAGCTCGGTTCCACGTCGACGCCACGCGTCTGGGTTGTGCCAACGCAA 891
ARR       ATCT-TGGCGCCGAGCTCGGTTCCACGTCGACGCCACGCGTCTGGGTTGTGCCAACGCAA 875
CCH       ATCTGTGGCGCCGAGCTCGGTTCCACGTCGACGCCACGCGTCTGGGTTGTGCCAACGCAA 890
CFT-1     ATCT-TGGCGCCGAGCTCGGTTCCACGTCGACGCCACGCGTCTGGGTTGTGCCAACGCAA 878
CFT-2     ATCT-TGGCGCCGAGCTCGGTTCCACGTCGACGCCACGCGTCTGGGTTGTGCCAACGCAA 879
MIS-1     ATCTGTGGCGCCGAGCTCGGTTCCACGTCGACGCCACGCGTCTGGGTTGTGCCAACGCA- 893
MIS-2     ATCTGTGGCGCCGAGCTCGGTTCCACGTCGACGCCACGCGTCTGGGTTGTGCCAACGCA- 892
          **** *******************************************************

          +++  24-nt                                     36-nt
W23       CACGACCTCGGCGCCATAGCCTATGGCGCCGAGC-------------------------- 912
B73       CACGACCTCGGCGCCATAGCCTATGGCGCCGAGCTGTGTTAGCTCGGCGCCATAGCCTAT 951
ARR       CACGTCCTCGGCGCCATAGCCTATGGCGCCGAGC-------------------------- 909
CCH       CACGACCTCGGCGCCATAGCCTATAGCGCCGAGC-------------------------- 924
CFT-1     CACGACCTCGGCGCCATAGCCTATGGCGCCGAGC-------------------------- 912
CFT-2     CACGACCTCGGCGCCATAGCCTATGGCGCCGAGC-------------------------- 913
MIS-1     ----------------------TGGCGCCGAGC-------------------------- 904
MIS-2     ----------------------TGGCGCCGAGC-------------------------- 903
                                * *********

                                                               +
W23       ----------AAAGGGTCCAAAACTGCATTTAAAATTTTTTTAGGTCTAAACGTGATTTT 962
B73       GGCGCCGAGCAAAGGGTCCAAAACTGCATTTAAAATTTTTTTAGGTCTAAACGTGATTTT 1011
ARR       ----------AAAGGGTCCAAAACTGCATTTAAAATTTTTTTAGGTCTAAACGTGATTTT 959
CCH       ----------AAAGGGTCCAAAACTGCATTTAAAATTTTTTTAGGTCTAAACGTGATTTT 974
CFT-1     ----------AAAGGGTCCAAAACTGCATTTAAAATTTTTTTAGGTCTAAACGTGATTTT 962
CFT-2     ----------AAAGGGTCCAAAACTGCATTTAAAATTTTTTTAGGTCTAAACGTGATTTT 963
MIS-1     ----------AAAGGGTCCAAAACTGCATTTAAAATTTTTTTAGGTCTAAACGTGATTTT 954
MIS-2     ----------AAAGGGTCCAAAACTGCATTTAAAATTTTTTTAGGTCTAAACGTGATTTT 953
                    **************************************************

                                           19-nt     +
W23       ACTTCTGTTTAAGGGCTAAAATACAAAAAAATTCGGTCGGTCCGTAACGTGCACTCTGCAC 1022
B73       ACTTCTGTTTAAGG-CTAAAATACAA------------------ACGTGCACTCTGCAC 1051
ARR       ACTTCTGTTTAAAGGCTAAAATACAAAAAAATTCGGTCGGTCCGTAACGCGCACTCTGCAC 1019
CCH       ACTTCTGTTTAAGGGCTAAAATACAA------------------ACGTGCACTCTGCAC 1015
CFT-1     ACTTCTGTTTAAAGGCTAAAATACAAAAAAATTCGGTCGGTCCGTAACGTGCACTCTGCAC 1022
CFT-2     ACTTCTGTTTAAAGGCTAAAATACAAAAAAATTCGGTCGGTCCGTAACGTGCACTCTGCAC 1023
MIS-1     ACTTCTGTTTAAGGGCTAAAATACAAAAAAATTCGGTCGGTCCGTAACGTGCACTCTGCAC 1014
MIS-2     ACTTCTGTTTAAGGGCTAAAATACAAAAAAATTCGGTCGGTCCGTAACGTGCACTCTGCAC 1013
          ************ * ***********                  *** ***********
```

```
                                            +                  18-nt
                                                          <---------------->
W23       TCTACTAAGCGCTAGTGTACGTACGTACGTACTCCGTCCGCTGCTATATTATGGCCGGCC 1082
B73       TCTACTAAGCGCTAGTGTACGTACGTACGTACTCCGTCCGCTGCTATATTATGGCCGGCC 1111
ARR       TCTACTAAGCGCTAGTGTACGTACGTACGTACTCCGTCCGCTGCTATATTATGGCCGGCC 1079
CCH       TCTACTAAGCGCTAGTGTACGTACGTACGTACTCCGTC------------------GGCC 1057
CFT-1     TCTACTAAGCGCTAGTGTACGTACGTACGTACTCCGTCCGCTGCTATATTATGGCCGGCC 1082
CFT-2     TCTACTAAGCGCTAGTGTACGTACGTACGTACTCCGTCCGCTGCTATATTATGGCCGGCC 1083
MIS-1     TCTACTAAGCGCTAGTGTACGTACGTACGTGCTCCGTCCGCTGCTATATTATGGCCGGCC 1074
MIS-2     TCTACTAAGCGCTAGTGTACGTACGTACGTGCTCCGTCCGCTGCTATATTATGGCCGGCC 1073
          ****************************** *******                  ****

                         +1   5'UTR
                              ------->
W23       GTGGCGTGCCCTCTCTAGCCAGCACAGCACAC 1114
B73       GTGGCGCGCCCTCTCTAGCCAGCACAGCACAC 1143
ARR       GTGGCGTGCCCTCTCTAGCCAGCACAGCACAC 1111
CCH       GTGGCGTGCCCTCTCTAGCCAGCACAGCACAC 1089
CFT-1     GTGGCGTGCCCTCTCTAGCCAGCACAGCACAC 1114
CFT-2     GTGGCGTGCCCTCTCTAGCCAGCACAGCACAC 1115
MIS-1     GTGGCGTGCCCTCTCTAGCCAGCACAGCACAC 1106
MIS-2     GTGGCGTGCCCTCTCTAGCCAGCACAGCACAC 1105
          ****** *************************
```

B

Distal enhancer (~600-bp)

```
B73   AAGCGAGGTGTGGAATGGGGAAGGCAGACGATGAGGAGAGCTCGATCCATCGCCCAACCC 60
W23   AAGCGAGGTGTGGAATGGGGAAGGCAGACGATGAGGAGAGCTCGATCCATCGCCCAACCC 60
ARR   AAGCGAGGTGTGGAATGGGGAAGGCAGACGATGAGGAGAGCTCGATCCATCGCCCAACCC 60
CCH   AAGCGAGGTGTGGAATGGGGAAGGCAGACGATGAGGAGAGCTCGATCCATCGCCCAACCC 60
CFT   AAGCGAGGTGTGGAATGGGGAAGGCAGACGATGAGGAGAGCTCGATCCATCGCCCAACCC 60
CNC   AAGCGAGGTGTGGAATGGGGAAGGCAGACGATGAGGAGAGCTCGATCCATCGCCCAACCC 60
MIS   AAGCGAGGTGTGGAATGGGGAAGGCAGACGATGAGGAGAGCTCGATCCATCGCCCAACCC 60
      ************************************************************

        ++++
B73   CAACCTATGTGGTTTGTTGCCTGCTCCCACTTTGTCTTGCCATCCATGTGTCGGCTACTG 120
W23   CAACCTATGTGGTTTGTTGCCTGCTCCCACTTTGTCTTGCCATCCATGTGTCGGCTACTG 120
ARR   CAACCTATGTGGTTTGTTGCCTGCTCCCACTTTGTCTTGCCATCCATGTGTCGGCTACTG 120
CCH   CAACCTATGTGGTTTGTTGCCTGCTCCCACTTTGTCTTGCCATCCATGTGTCGGCTACTG 120
CFT   CAACCTATGTGGTTTGTTGCCTGCTCCCACTTTGTCTTGCCATCCATGTGTCGGCTACTG 120
CNC   CAACCTATGTGGTTTGTTGCCTGCTCCCACTTTGTCTTGCCATCCATGTGTCGGCTACTG 120
MIS   CAACCTATGTGGTTTGTTGCCTGCTCCCACTTTGTCTTGCCATCCATGTGTCGGCTACTG 120
      ************************************************************

                              *            **         **
B73   CTCCCTTGCGTAATTATTATTCAAGTTTGGCGATCCAAGAGACCCCAAGATATGTGTGTG 180
W23   CTCCCTTGCGCAATTATTATTCAAGTTTGGCGATCCAAGAGACCCCAAGATATGTGTGTG 180
ARR   CTCCCTTGCGCAATTATTATTCAAGTTTGGCGATCCAAGAGCCCCCAAGATATGTGTGTG 180
CCH   CTCCCTTGCGCAATTATTATTCAAGTTTGGCGATCCAAGAGACCCCAAGATATGTGTGTG 180
CFT   CTCCCTTGCGCAATTATTATTCAAGTTTGGCGATCCAAGAGCCCCCAAGATATGTGTGTG 180
CNC   CTCCCTTGCGCAATTATTATTCAAGTTTGGCGATCCAAGAGCCCCCAAGATATGTGTGTG 180
MIS   CTCCCTTGCGCAATTATTATTCAAGTTTGGCGATCCAAGAGCCCCCAAGATATGTGTGTG 180
      ********** ****************************** ******************

B73   CTCGCTCGCTCGCTCGCTGCCGTCGCGTGGGTCTTGGTTCAGATGGCCAAATAATTGCAG 240
W23   CTCGCTCGCTCGCTCGCTGCCGTCGCGTGGGTCTTGGTTCAGATGGCCAAATAATTGCAG 240
ARR   CTCGACTGCTCGCTCGCTGCCGTCGCGTGGGTCTTCGTTCAGATGGCCAAATAATTGCAG 240
CCH   CTCGCTCGCTCGCTCGCTGCCGTCGCGTGGGTCTTGGTTCAGATGGCCAAATAATTGCAG 240
CFT   CTCGACTGCTCGCTCGCTGCCGTCGCGTGGGTCTTCGTTCAGATGGCCAAATAATTGCAG 240
CNC   CTCGACTGCTCGCTCGCTGCCGTCGCGTGGGTCTTCGTTCAGATGGCCAAATAATTGCAG 240
MIS   CTCGACTGCTCGCTCGCTGCCGTCGCGTGGGTCTTCGTTCAGATGGCCAAATAATTGCAG 240
      ****  **************************** ************************
```

```
B73    GGAGGGACCAATCGCCGCTGCAGCAGTGCCCAGTGAGTGCCACCACCGCGCGCTTGTCTT 300
W23    GGAGGGACCAATCGCCGCTGCAGCAGTGCCCAGTGAGTGCCACCACCACGCGCTTGTCTT 300
ARR    GGAGGGACCAATCGCCGCTGCAGCAGTGCCCAGTGAGTGGTGCCACCACGCGCTTGTCTT 300
CCH    GGAGGGACCAATCGCCGCTGCAGCAGTGCCCAGTGAGTGCCACCACCACGCGCTTGTCTT 300
CFT    GGAGGGACCAATCGCCGCTGCAGCAGTGCCCAGTGAGTGGTGCCACCACGCGCTTGTCTT 300
CNC    GGAGGGACCAATCGCCGCTGCAGCAGTGCCCAGTGAGTGGTGCCACCGCGCGCTTGTCTT 300
MIS    GGAGGGACCAATCGCCGCTGCAGCAGTGCCCAGTGAGTGGTGCCACCACGCGCTTGTCTT 300
       ***************************************  ***** ************

B73    GTCAGCTTGCGGAGAGCCACCACATGCTTCCCACATGATGAGCCCCAGGCAGGCTGACGA 360
W23    GTCAGCTTGCGGAGAGCCACCACATGCTTCCCACATGATGAGCCCCAGGCAGGCTGACGA 360
ARR    GTCAGCTTGCGGAGAGCCACCACATGCTTCCCACATGATGAGCCCCAGGCAGGCTGACGA 360
CCH    GTCAGCTTGCGGAGAGCCACCACATGCTTCCCACATGATGAGCCCCAGGCAGGCTGACGA 360
CFT    GTCAGCTTGCGGAGAGCCACCACATGCTTCCCACATGATGAGCCCCAGGCAGGCTGACGA 360
CNC    GTCAGCTTGCGGAGAGCCACCACATGCTTCCCACATGATGAGCCCCAGGCAGGCTGACGA 360
MIS    GTCAGCTTGCGGAGAGCCACCACATGCTTCCCACATGATGAGCCCCAGGCAGGCTGACGA 360
       ************************************************************

B73    CGTCTCACCGGCTCACACCTCCTCCTCCGTCCTCAAAACCAAAGCGTTGCGTTGCATGCT 420
W23    CGTCTCACCGGCTCACACCTCCTCCTCCGTCCTCAAAACCAAAGCGTTGCGTTGCATGCT 420
ARR    CGTCTCACCGGCTCACACCTCCTCCTCCGTCCTCAAAACCAAAGCGTTGCGTTGCATGCT 420
CCH    CGTCTCACCGGCTCACACCTCCTCCTCCGTCCTCAAAACCAAAGCGTTGCGTTGCATGCT 420
CFT    CGTCTCACCGGCTCACACCTCCTCCTCCGTCCTCAAAACCAAAGCGTTGCGTTGCATGCT 420
CNC    CGTCTCACCGGCTCACACCTCCTCCTCCGTCCTCAAAACCAAAGCGTTGCGTTGCATGCT 420
MIS    CGTCTCACCGGCTCACACCTCCTCCTCCGTCCTCAAAACCAAAGCGTTGCGTTGCATGCT 420
       ************************************************************

                    +++
B73    TTGTTTGCTTCCGCACATCGACGGTCATATGCATGGATGCATGGGTGATCGGTGACGTAG 480
W23    TTGTTTGCTTCCGCACATCGACGGTCATATGCATGGATGCATGGGTGATCGGTGACGTAG 480
ARR    TTGTTTGCTTCCGCACGTCGACGGCCATATGCATGCATGCATGGGTGATCGGTGACGTAG 480
CCH    TTGTTTGCTTCCGCACATCGACGGTCATATGCATGGATGCATGGGTGATCGGTGACGTAG 480
CFT    TTGTTTGCTTCCGCACGTCGACGGCCATATGCATGCATGCATGGGTGATCGGTGACGTAG 480
CNC    TTGTTTGCTTCCGCACGTCGACGGCCATATGCATGCATGCATGGGTGATCGGTGACGTAG 480
MIS    TTGTTTGCTTCCGCACGTCGACGGCCATATGCATGCATGCATGGGTGATCGGTGACGTAG 480
       **************** ******* ********** ************************

B73    CAGCGGCTTCTCGGTGTGTGTCGTCGCTAGCTGGCTAGTGTGCGGTCGAGTTTGTTCGTG 540
W23    CAGCGGCTTCTCGGTGTGTGTCGTCGCCAGCTGGCCAGTGTGCGGTCGAGTTTGTTCGTG 540
ARR    CAGCGGCTTCTCGGTGTGTGTCGTCGCTAGCTGGCCAGTGTGCGGTCGAGTTTGTTCGTG 540
CCH    CAGCGGCTTCTCGGTGTGTGTCGTCGCTAGCTGGCCAGTGTGCGGTCGAGTTTGTTCGTG 540
CFT    CAGCGGCTTCTCGGTGTGTGTCGTCGCTAGCTGGCCAGTGTGCGGTCGAGTTTGTTCGTG 540
CNC    CAGCGGCTTCTCGGTGTGTGTCGTCGCTAGCTGGCCAGTGTGCGGTCGAGTTTGTTCGTG 540
MIS    CAGCGGCTTCTCGGTGTGTGTCGTCGCTAGCTGGCCAGTGTGCGGTCGAGTTTGTTCGTG 540
       *************************** ******* ************************

B73    CTAATTAAACGAGGAGAAATCATTGTTTGCAGGCGCCACCTGATGATCGAAGCGGATTAC 600
W23    CTAATTAAACGAGGAGAAATCATTGTTTGCAGGCGCCACCTGATGATCGAAGCGGATTAC 600
ARR    CTAATTAAACGAGGAGAAATCATTGTTTGCAGGCGCCACCTGATGATCGAAGCGGATTAC 600
CCH    CTAATTAAACGAGGAGAAATCATTGTTTGCAGGCGCCACCTGATGATCGAAGCGGATTAC 600
CFT    CTAATTAAACGAT---AAATCATTGTTTGCAGGCGCTACCTGATGATCGAAGCGGATTAC 597
CNC    CTAATTAAACGAT---AAATCATTGTTTGCAGGCGCTACCTGATGATCGAAGCGGATTAC 597
MIS    CTAATTAAACGAGGAGAAATCATTGTTTGCAGGCGCCACCTGATGATCGAAGCGGATTAC 600
       ************     ******************** ***********************
```

```
B73     TCACCGCCCTCGGCTGTTCGATGCCATCATAAT 633
W23     TCACCGCCCTCGGCTGTTCGATGCCATCATAAT 633
ARR     TCACCGCCCTCGGCTGTTCGATGCCATCATAAT 633
CCH     TCACCGCCCTCGGCTGTTCGATGCCATCATAAT 633
CFT     TCACCGCCCTCGGCTGTTCGATGCCATCATAAT 630
CNC     TCACCGCCCTCGGCTGTTCGATGCCATCATAAT 630
MIS     TCACCGCCCTCGGCTGTTCGATGCCATCATAAT 633
        *********************************
```

C

INTRON I

```
                                                +++
W23-I1    GTAAACCAAAGCCGGCCGCGCGCCATGCATCGCCACGTAGCATCAATCTCCGATCCATGC 60
B73-I1    GTAAACCAAAGCCGGCCGCGCGCCATGCATCGCCACGTAGCATCAATCTCCGATCCATGC 60
ARR-I1    GTAAACCAAAGCCGGCCGCGCGCCATGCATCGCCACGTAGCATCAATCTCCGATCCATGC 60
CCH-I1    GTAAACCAAAGCCGGCCGCGCGCCATGCATCGCCACGTAGCATCAATCTCCGATCCATGC 60
CFT-I1    GTAAAACAAAGCCGGCCGCCCGCCATGCATCGGCACGTTCCATCAATCTCCGATCCATGC 60
MIS-I1    GTAAACCAAACCCGGCCGCGCGCCATGCATCGCCACGTAGCATCAATCTCCGATCCATGC 60
          *****.**** ******** ************ *****: ********************

W23-I1    ATATATGAGCTTCTTCTTCGTCGCCGTCGTCGTTCTTAGCTAGTTAGGACGCGCATGCAG 120
B73-I1    ATATATGAGCTTCTTCTTCGTCGCCGTCGTCGTTCTTAGCTAGTTAGGACGCGCATGCAG 120
ARR-I1    ATATATGAGCTTCTTCTTCGTCGCCGTCGTCGTTCTTAGCTAGTTAGGACGCGCATGCAG 120
CCH-I1    ATATATGAGCTTCTTCTTCGTCGCCGTCGTCGTTCTTAGCTAGTTAGGACGCGCATGCAG 120
CFT-I1    ATATATGAGCTTCTTCTTCGTCGCCGTCGTCGTTCTTAGCTAGTTAGGACGCGCATGCAG 120
MIS-I1    ATATATGAGCTTCTTCTTCGTCGCCGTCGTCGTTCTTAGCTAGTTAGGACGCGCATGCAG 120
          ************************************************************
```

Supplemental figure 1

Multiple sequence alignment CLUSTAL 2.1 (A) Proximal enhancer; (B) Distal enhancer; (C) Intron I.

UVBox core (*, CAAG) UVBox (5-nt, **, CCAAG or CAAGG); ACE^{ANAC13} (+, GCTACGTGTC, core underlined), MRE^{ANAC13} (++, CCAAACCTTCTT), ACE^{CHS} core (+++, CACGT), MRE^{CHS} core (++++, ACCTA).
