# Supplementary figures and images for: Analysis of the P1 promoter in response to UV-B radiation in allelic variants of high-altitude maize
Source: BMC Plant Biol. 2012 Jun 15;12:92. doi: 10.1186/1471-2229-12-92 (PMC3489873; doi:10.1186/1471-2229-12-92)

Figure S2

A

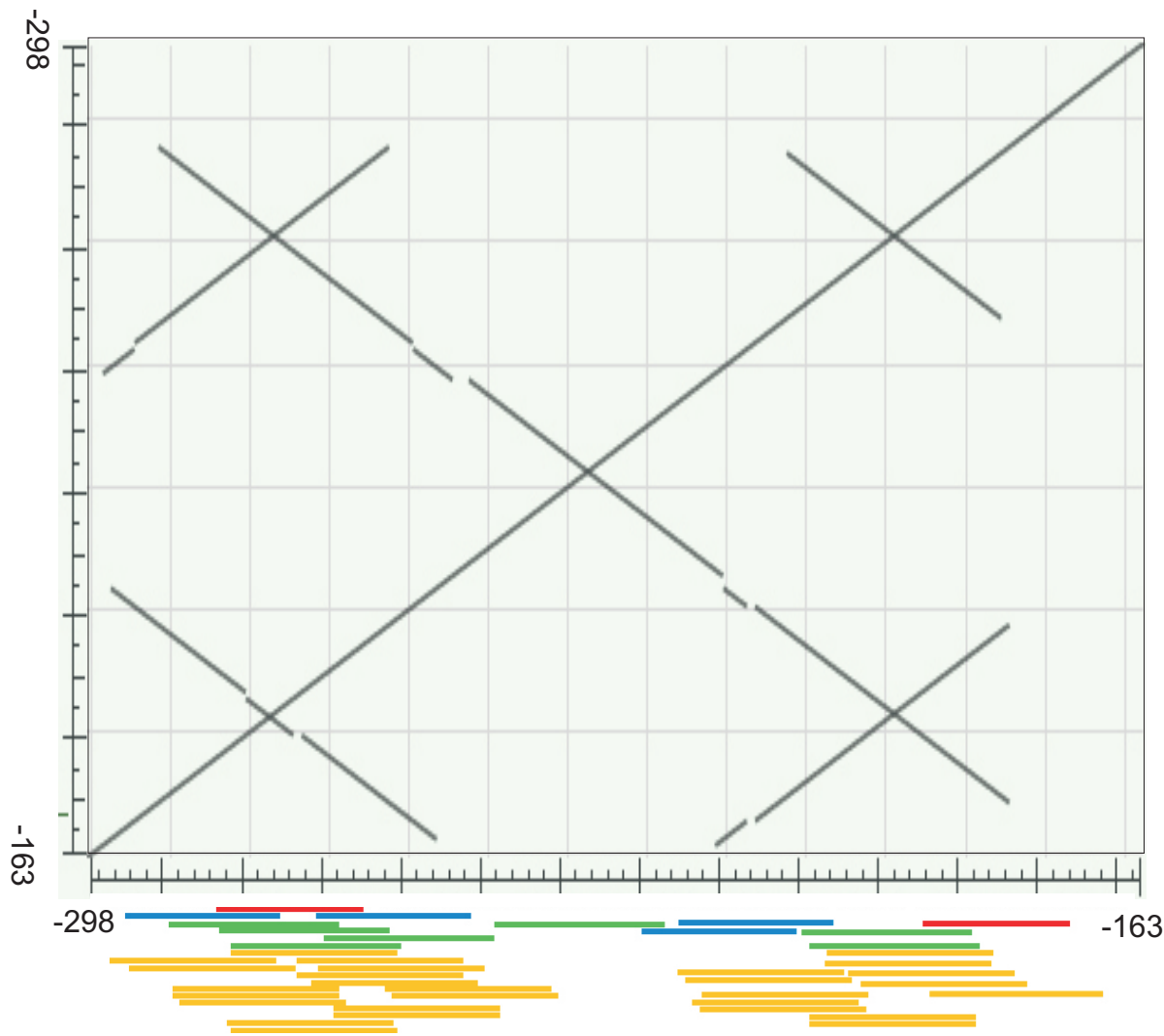

Supplement: Additional file 2 — Table S1. List of primers sequences. Table S2. Nucleotide diversity in the p1 alleles. [file 1471-2229-12-92-S2.pdf]

# Supplemental Figure 3

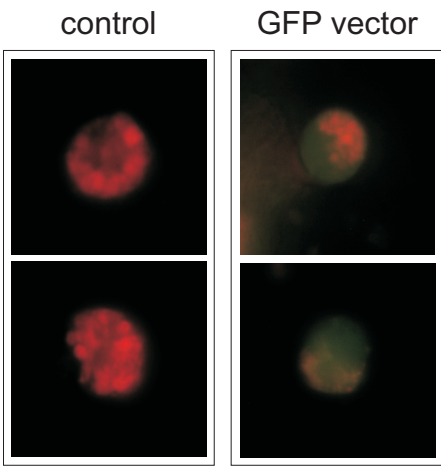

Supplement: Additional file 3 — Figure S2. Dot matrix plot representing direct and inverted repeats of the sequence (108 bps) corresponding to the expanded region of the proximal promoter described in Figure 5A. Numbers indicate position relative to SST of P1 on x and y axis. [file 1471-2229-12-92-S3.pdf]
