# Supplementary material for: Analysis of the P1 promoter in response to UV-B radiation in allelic variants of high-altitude maize
Source: BMC Plant Biol. 2012 Jun 15;12:92. doi: 10.1186/1471-2229-12-92 (PMC3489873; doi:10.1186/1471-2229-12-92)
Supplement: Additional file 4 — Figure S3. Integrity of protoplasts irradiated with UV-B after 8 h of exposition. Left panel: protoplast not expressing GFP (control), right panel: protoplasts transformed with GFP. [file 1471-2229-12-92-S4.pdf]

Table S1. List of primers sequences

Primer name	Sequence
P1-distal-fw	AAGCGAGGTGTGGAATGGGG
P1-distal-rv	ATGATGGCATCGAACAGCCG
P1-proximal-fw	CATATATCGGCCTGGGATTG
P1-proximal-rv	GTGTGCTGTGCTGGCTAGAG
pGEMTe-*Not*I-fw	CCATGGCGGCCGCGGGAATT
pGEMTe-*Kpn*I-rv	AGGCGGTACCGAATTCACTAGTGATA
P1-1070-fw	CCTGCTCCCACTTTGTCTTG
P1-1421-rv	GAAGCAAACAAAGCATGCAA
P1-intron I-fw	AAACCATGGGTCGCTGCCCAAGAATGCAG
P1-intron I-rv	AAACTCGAGCTTGCCGCACCGGAGCAGGC
ACT-1fw	CTTCGATGCCCAGCAAT
ACT-1rv	CGGAGAATAGCATGAGGAAG
C2-fw	CCGTCCAACTGACCTAACC
C2-rv	GAGCTAGCGATCGAGCTG

Underlined sequence indicates enzyme restriction site.

Table S2. Nucleotide diversity in the *p1* alleles

	n **Regions sites**	**N° of silent sites**	**N° of**
**polymorphic**			
proximal promoter	8	1172	30
distal enhancer	7	633	17
1st intron	6	120	6

	**π Nucleotide diversity, Pi**	**Θ Theta from Eta** (per sequence)	**θ Theta from Eta** (per site)	**Tajima's D**
proximal promoter	0.00851	11.57025	0.01111	-1.24298, NS
distal enhancer	0.01361	6.93878	0.01101	1.31014, NS
1st intron	0.01667	2.62774	0.02190	-1.36732, NS

Nucleotide diversity was estimated on the *p1* haplotypes. NS, not significant. Results from DNA sequences polymorphism program (DNAsp5.0) (Librado and Rozas 2009).
